# Advances in the Understanding of Akt Signaling in Cancers and the Potential of Inhibiting Akt-Driven Tumors Using Small Molecule Inhibitors: An Overview

**DOI:** 10.3390/cancers18040578

**Published:** 2026-02-10

**Authors:** Jamuna Bai Aswathanarayan, Rimshia Naaz, Shalini H. Doreswamy, Medha Karnik, Sathish Kumar, Asha Sreenivasan, Arati Sharma, SubbaRao V. Madhunapantula

**Affiliations:** 1 Department of Microbiology, School of Life Sciences Mysuru, JSS Academy of Higher Education & Research (JSS AHER), Mysore 570015, Karnataka, India; jamunabhounsle@jssuni.edu.in; 2 Department of Physiology, JSS Medical College, JSS Academy of Higher Education & Research (JSS AHER), Mysore 570015, Karnataka, India; rimshiannaaz@jssuni.edu.in; 3 Center of Excellence in Molecular Biology and Regenerative Medicine (CEMR) Laboratory, Department of Biochemistry, JSS Medical College, JSS Academy of Higher Education & Research (JSS AHER), Mysore 570015, Karnataka, India; medhakarniksr@jssuni.edu.in; 4 Division of Nanoscience and Technology, School of Life Sciences Mysuru, JSS Academy of Higher Education & Research (JSS AHER), Mysore 570015, Karnataka, India; shalinihd@jssuni.edu.in (S.H.D.); asha.srinivasan@jssuni.edu.in (A.S.); 5 Division of Molecular Biology, School of Life sciences Mysuru, JSS Academy of Higher Education & Research (JSS AHER), Mysore 570015, Karnataka, India; hssathish@jssuni.edu.in; 6 Department of Molecular and Precision Medicine, Center for Cannabis and Natural Product Pharmaceuticals (CCNPP), Penn State Cancer Institute, Hershey, PA 17033, USA; asharma@pennstatehealth.psu.edu; 7 Special Interest Group in Cancer Biology and Cancer Stem Cells (SIG-CBCSC), JSS Medical College, JSS Academy of Higher Education & Research (JSS AHER), Mysore 570015, Karnataka, India

**Keywords:** protein kinase-B, Akt, breast cancer, melanoma, PTEN, PI3K, PRAS40

## Abstract

The phosphatidylinositol-3-kinase (PI3K)/Akt signaling pathway is a central regulator of cellular metabolism, survival, and proliferation and is frequently dysregulated in cancer. Since the identification of protein kinase B (Akt) in 1996, extensive research has established its critical role in tumor initiation, progression, and therapeutic resistance, making Akt an attractive target for anticancer drug development. Although numerous inhibitors targeting the PI3K/Akt pathway have been developed, their clinical success has been limited due to inadequate isoform specificity and unfavorable toxicity profiles. These limitations have prompted increasing interest in identifying Akt-selective inhibitors from natural sources, particularly microbial metabolites. Recent in vitro and in vivo studies demonstrate that several microbial-derived compounds effectively modulate PI3K/Akt signaling and suppress key cancer hallmarks, including proliferation, angiogenesis, and metastatic potential. Nevertheless, further studies are required to define Akt isoform specificity, evaluate selectivity against closely related kinases, and validate therapeutic efficacy in relevant preclinical models, including patient-derived xenografts. In addition, the development of robust purification and optimization strategies remains essential to enable the reliable isolation and translational advancement of these bioactive metabolites. This review summarizes Akt structure, function, and key regulatory motifs relevant to pharmacological targeting and critically examines microbial-derived inhibitors of the PI3K/Akt pathway and their mechanisms of action. Representative compounds discussed include Bostrycin, Anthracycline analogs, Wentilactone A, Thiocoraline, Iturin A, SZ-685C, Isebromoamide B, Xyloketal B, and Demethoxyfumitremorgin C. Collectively, this review highlights the therapeutic potential of microbial natural products while outlining current challenges and future directions for developing selective Akt-targeted anticancer therapies.

## Simple Summary

The PI3K/Akt pathway is a key regulator of cancer cell survival and proliferation, but current inhibitors show limited clinical success due to poor isoform specificity and toxicity. These limitations have fueled growing interest in discovering Akt-selective inhibitors derived from natural sources, such as plants, animals and microbes. Notably, microbial-derived natural products have emerged as promising Akt-selective inhibitors, demonstrating suppression of major cancer hallmarks in preclinical studies. Collectively, this review highlights the oncogenic potential of Akt, its structure and regulation, isoform-specific functions, the critical role of mutations and posttranslational modifications, a summary of the small molecular inhibitors of Akt from plants, animals and microbes and their mechanisms, and discusses challenges and future directions for developing selective anticancer therapies.

## 1. Introduction

### 1.1. Oncogenic Potential of Akt

**Figure 1:**
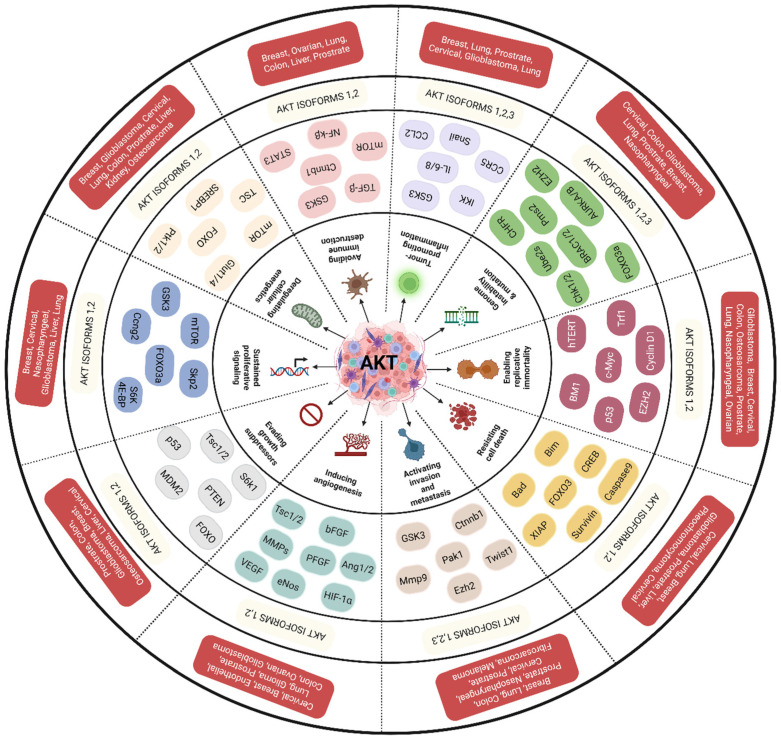
AKT Signaling as a Central Regulator of the Hallmarks of Cancer. This schematic illustrates the central role of AKT signaling in regulating the hallmarks of cancer. The inner ring depicts the major hallmarks of cancer influenced by AKT activation, which includes (a) sustained proliferative signaling, (b) evasion of growth suppressors, (c) resistance to cell death, (d) enabling replicative immortality, (e) induction of angiogenesis, (f) activating invasion and metastasis, (g) tumor-promoting inflammation, (h) reprogramming of energy metabolism, (i) avoidance of immune destruction and (j) genetic instability and mutations. Surrounding each hallmark are representative downstream molecular targets and pathways modulated by AKT (e.g., mTOR, GSK3, FOXO, p53, MDM2, HIF-1α, VEGF, MMPs, EMT regulators, and apoptosis-related proteins), indicating how AKT signaling orchestrates these oncogenic processes. The outer ring denotes AKT isoforms (AKT1, AKT2, and AKT3) and their associations with specific cancer types, including breast, cervical, lung, colon, prostate, glioblastoma, liver, ovarian, and other malignancies. Therefore, targeting Akt by using isoform-specific inhibitors is a potentially viable strategy to control tumor growth.

**Figure 2:**
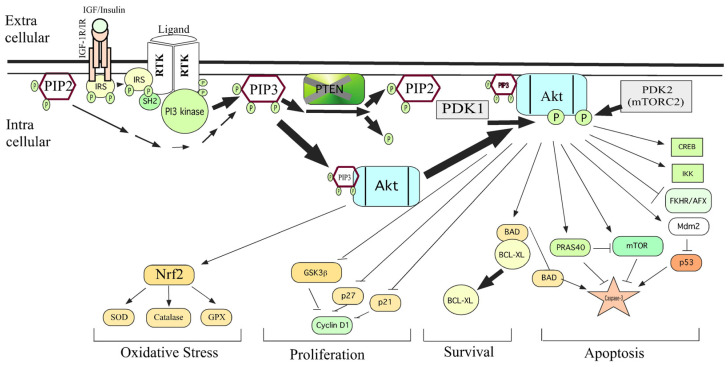
Canonical PI3K–AKT–mTOR Signaling Pathway and Its Role in Cancer-Related Cellular Processes. This figure depicts the canonical PI3K–AKT–mTOR signaling cascade initiated by extracellular growth factors, hormones, and cytokines. Binding of these ligands to receptor tyrosine kinases at the cell membrane leads to recruitment and phosphorylation of IRS-1 and activation of PI3K, which converts PIP2 to PIP3. The tumor suppressor PTEN negatively regulates this step by dephosphorylating PIP3. Accumulation of PIP3 facilitates membrane recruitment of PDK1 and AKT/PKB, resulting in AKT phosphorylation at Thr308 and Ser473 (the latter mediated by mTORC2), leading to full AKT activation. Activated AKT phosphorylates multiple downstream substrates that regulate various hallmarks of cancer cells. Vertical and horizontal inhibition of Akt pathway members is an attractive strategy to retard tumor growth. Studies are warranted to test these possibilities.

The protein kinase-B (PKB) also known as Akt (for AKR mouse strain Transforming or Thymoma) is one of the key kinases well-explored not only for its functions in insulin signaling but also for its ability to control the survival of cells [1]. Along with its regulator, i.e., phosphotidylinositol-3-kinase (PI3K), the Akt controls various hallmark features of cancer cells and thereby promotes tumor progression and enhances resistance of cancer cells to drugs (Figure 1) [2]. Dysregulated PI3K-Akt signaling has been reported in various cancers (Figure 2) [2,3]. Unusual activation of PI3K/Akt signaling occurs due to (a) genetic aberrations such as deletion of negative regulators such as PTEN; (b) gain-of-function mutations in Akt (such as E40K) or proteins that activate Akt (such as PI3K); and (c) overexpression of the Akt due to increased copy number [4]. Overexpression of Akt in normal cells could transform them in to tumorigenic ones, demonstrating its oncogenic potential [5]. Further evidences have emerged from knockdown experiments wherein targeted inhibition of Akt expression reduced the proliferation and survival of tumor cells [6,7]. Subsequently, studies have shown that it is the kinase activity of Akt, but not just the protein itself, which is responsible for aggressive behavior of tumor cells [8]. In essence, elevated functionally active Akt is responsible for tumor cells’ growth, drug resistance and metastatic spread. Therefore, targeted downregulation of Akt is a viable strategy to retard tumor growth, and studies are immediately needed to identify effective pharmacological agents that can inhibit Akt activity [4]. This review not only covers the structural and functional properties of Akt but also provides an in-depth understanding of how the post-translational modifications impact the Akt expression and function. Furthermore, we have provided a detailed note on how Akt controls various hallmark features of cancer cells. Finally, a detailed description of Akt inhibitors and their current status in clinical development has been discussed.

### 1.2. Akt Exhibits Isoform-Specific Functions in Cancer Cells

**Figure 3:**
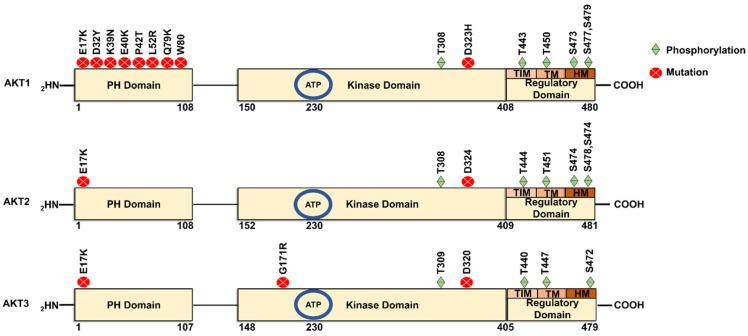
Domain Architecture, Phosphorylation Sites, and Cancer-Associated Mutations in AKT Isoforms (AKT1, AKT2, and AKT3). This figure illustrates the structural organization of the three human AKT isoforms—AKT1, AKT2, and AKT3—highlighting their conserved domains, key phosphorylation sites, and recurrent cancer-associated mutations. Each isoform comprises an N-terminal pleckstrin homology (PH) domain, a central kinase domain containing the ATP-binding site, and a C-terminal regulatory domain that includes the turn motif (TM/TIM) and hydrophobic motif (HM). Green diamonds indicate major activating phosphorylation sites, including Thr308/309 within the activation loop and Ser473/474/472 (respectively for Akt1, Akt2 and Akt3) within the hydrophobic motif, which are essential for full AKT activation. Red crossed circles denote hotspot somatic mutations reported in cancers, which can lead to constitutive membrane localization or altered kinase activity. Residue numbering reflects isoform-specific differences in length and sequence.

Akt is a serine/threonine kinase in the phosphatidyl-inositole-3 kinase (PI3K) pathway [9]. Its major functions include regulation of cell proliferation, survival, and metabolism [10]. Structurally, Akt exists in three isoforms, viz., Akt1 (PKBα), Akt2 (PKBβ), and Akt3 (PKBγ), which are coded by different genes (Figure 3). The genes encoding Akt1, Akt2 and Akt3 are located respectively on the long arm of chromosomes 14 (14q32), 19 (19q13) and 1 (1q13) [10]. A very high degree of homology has been observed among the three isoforms (82% homology between Akt1 and Akt2; 83% between Akt1 and Akt3; and 77% between Akt2 and Akt3), suggesting the possibility of overlapping functions and difficulty in designing selective isoform-specific inhibitors. But, despite having high sequence homology, there are certain differences in amino acid sequence which contribute to isoform-specific functions (Figure 3) [11,12] These isoform-specific functions play significant roles not only in the pathogenesis of diseases but also provide opportunities to design selective pharmacological agents to mitigate the activity [11].

Akt1 and Akt2 are expressed in the majority of cell types, whereas Akt3 is generally present in neural and skin cells [13]. Activation of Akt isoforms leads to oncogenesis as observed in carcinomas of the breasts, ovaries, pancreas, and prostate [14]. Whereas Akt1 regulates proliferation and growth of tumor cells, and enhances tumor initiation through the suppression of apoptosis, Akt2 regulates cell migration, invasiveness and metastasis. Studies related to Akt3 are very limited, hence its isoform-specific functions are yet to emerge [12,15]. Stahl et al., have reported selective activation of Akt3 but not Akt2 or Akt1 in malignant melanoma tissues compared to normal cell lines and adjacent non-tumor skin biopsies [16]. Overexpressed Akt3 has been shown to promote melanoma cells growth by phosphorylating proline-rich Akt substrate of 40KDa (PRAS40), glycogen synthase kinase (GSK3) and mammalian target of rapamycin (mTOR). Targeted downregulation of Akt3 but not Akt2 and Akt1 reduced the viability of melanoma cells while sensitizing them to chemotherapeutic agents such as Staurosporine. Authors of these studies have concluded that Akt3 is a viable therapeutic target in melanomas. But, it is unknown whether Akt3 alone is contributing to the melanoma tumors’ development and metastatic spread. Therefore, it is critical to thoroughly understand the isoform-specific functions of Akt. Moreover, to date, no pharmacological agents specifically targeting Akt3 have been identified, warranting additional investigations.

### 1.3. Structural Features That Determine the Oncogenic Potential of Akt

The domain structure of Akt isoforms is depicted in Figure 3. Studies using chimeric constructs and site-directed mutagenesis experiments have identified key structural features that are responsible for the oncogenic potential of Akt. For instance, chimeric constructs expressing the PH domain of Akt1 and catalytic and regulatory domains of Akt2 suggested the involvement of the PH domain in the translocation of Akt protein to the cell membrane [17]. Studies have also shown that truncated Akt lacking the PH domain is functionally inactive [18]. Therefore, pharmacological agents that bind to the PH domain and retard the translocation are viable candidates for inhibiting the Akt activity. Utilizing this knowledge, Gagic. et al. 2020 designed a set of non-lipid PH domain-binding inhibitors by applying in silico methods, synthesizing and characterizing a set of derivatives (Compounds 2 to 6) for safety and efficacy in vitro and in vivo [19]. Authors of this study identified NSC 348900 as the lead molecule with a binding affinity (K_D_) of 1.4µM to the PH domain of Akt1. All other compounds exhibited poor binding affinities to the PH domain and hence were not studied in detail in this investigation. In vitro studies against colorectal carcinoma cell line HT29 have demonstrated the potential of Compound 1 and its derivatives (Compounds 2 to 6) for inhibiting Akt activity. Despite encouraging results of these compounds in vitro, the in vivo efficacy studies failed to demonstrate tumor growth inhibition, which could be due to poor pharmacokinetic and dynamic properties. Future studies should focus more on further optimization of these compounds to improve the in vivo safety and efficacy.

Zhang M. et al., 2020 had isolated a compound called “Swertiamarin” (SW) from *Flos Lanicerae japonicae* and tested its efficacy for binding to the PH domain of Akt. Authors have shown that SW effectively binds to the PH domain of Akt and thereby reduces the inflammation by decreasing the pro-inflammatory cytokines TNF alpha, IL-8 and IL-6 [20]. However, its safety and efficacy for reducing the burden of tumors is yet to be evaluated. Very few studies have tested SW for retarding the tumor growth. A study showed that SW inhibits the proliferation of cervical cancer cells by inducing apoptotic cell death [21]. Although SW is known as a potent inhibitor of Akt, a few studies have also shown that it retards the proliferation of cells by inhibiting pErk and pMek. Additional studies are warranted to evaluate and determine its mechanisms of action and test whether the anti-cancer effects are mediated by the inhibition of Akt signaling or any other mechanisms involved. Furthermore, additional studies are needed to test this compound’s safety and efficacy in vivo.

### 1.4. Mutations That Activate Akt in Cancer Cells

Elevated expression and activity of Akt have been reported in several cancers [22]. Functionally active Akt confers resistance to chemotherapy by promoting the expression of drug export proteins while enhancing the enzymes that degrade pharmacological agents [23]. The Akt activity is regulated by intrinsic (within the gene/protein) and extrinsic (epigenetic and post-translational/protein–protein interaction) mechanisms. One of the intrinsic mechanisms is the presence of activity-enhancing mutations in the gene, which produces a hyper-active Akt [24]. One of the predominant somatic mutations in the Akt is E40K, in which the 40th amino acid, i.e., glutamic acid, is replaced by lysine. Akt containing the E40K mutation has been reported in carcinomas of breast (6–8%), brain (6%), and colon and rectum (2–6%). In addition to the E40K mutation, several other mutations in AKT have been reported to influence its oncogenic activity. Notably, the E17K mutation has been implicated in tumorigenesis by altering AKT function and promoting aberrant signaling [25]. Similarly, the well-characterized E17K hotspot mutation leads to constitutive activation of AKT and has been identified in breast, colorectal, and ovarian cancers [26]. Other variants, such as D23Y, H39N, P24T, L52R, Q79K, W80C and D323H, further highlight the diversity of AKT mutations and their potential impact on altered cellular signaling, cancer progression, drug resistance and therapeutic response (Figure 3).

### 1.5. Posttranslational Modifications Controlling the Activity of Akt in Cancer Cells

Post-translational modifications (PTMs) of proteins are covalent modifications that determine the physicochemical properties, spatial conformation, stability, cellular localization, and interactions with other proteins [27]. PTMs play important roles in regulating a variety of cellular functions and pathological processes [2,28,29,30]. Emerging research increasingly demonstrates that critical cellular processes, such as intracellular signaling, metabolic regulation, oxidative stress management and DNA repair are not solely dependent on protein abundance, but are instead significantly influenced by PTMs.

Although much attention has been given to the upstream activators of Akt, recent research highlights the significance of the PTMs of Akt in modulating its activity and, consequently, its role in oncogenesis [31]. Studies have shown that the activation of Akt in cancer cells is controlled by various PTMs, which include (a) phosphorylation of serine/threonine residues; (b) acylation of O-linked Glucosamine residues; and (c) modification(s) of lysine residues either by ubiquitination, SUMOylation and acetylation (Table 1). Among these PTMs, K63-linked ubiquitination is very critical for the activation of Akt as it facilitates the translocation to the cell membrane [22]. Deficiency of E3 ligases that are responsible for the K63-linked ubiquitination leads to tumor suppression by decreasing the activity of Akt. Therefore, a comprehensive understanding of PTMs that control the activity of Akt is likely to offer novel strategies for cancer therapy.

#### 1.5.1. Phosphorylation and Activation of Akt

**Figure 4:**
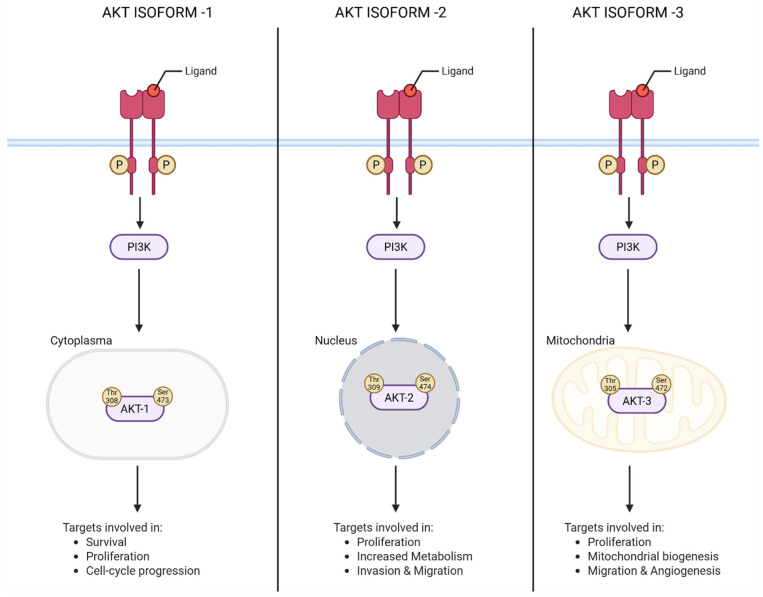
Isoform-Specific Activation, Subcellular Localization, and Functional Roles of AKT1, AKT2, and AKT3. This figure illustrates the isoform-specific activation and downstream functions of the three AKT isoforms following ligand-induced receptor tyrosine kinase signaling and PI3K activation. Upon ligand binding, receptor phosphorylation triggers PI3K signaling, leading to activation of distinct AKT isoforms that preferentially localize to different subcellular compartments. AKT1 is predominantly activated in the cytoplasm and undergoes phosphorylation at Thr308 and Ser473, where it regulates targets involved in cell survival, proliferation, and cell cycle progression. AKT2 preferentially localizes to the nucleus following phosphorylation at Thr309 and Ser474 and primarily regulates pathways associated with cellular proliferation, enhanced metabolism, invasion, and migration. AKT3 shows preferential localization to mitochondria and is activated by phosphorylation at Thr305 and Ser472. It controls targets linked to proliferation, mitochondrial biogenesis, migration, and angiogenesis. Differential expression and activation of these Akt isoforms vary from cancer to cancer, hence, prior knowledge about the isoform(s) involved in a particular cancer is essential for developing better therapies.

Phosphorylation remains the most extensively studied PTM regulating Akt activity, serving as a canonical mechanism for its activation and functional diversification in cancer [32]. Akt isoforms can be phosphorylated at 20 different sites, which contribute to distinct substrate specificities and functionality. The most phosphorylated sites in the three isoforms are threonine Thr-308, Thr-309, Thr-305 at the catalytic domain and serines Ser-473, Ser-474 and Ser-472 at regulatory domains of Akt1, Akt2, and Akt3, respectively [33] (Figure 4, Table 1). Phosphorylation of Thr-308 is mediated by phosphoinositide-dependent kinase-1 (PDK1), but the phosphorylation of Ser 473 is catalyzed by PDK2, which was later identified as the mechanistic Target of Rapamycin Complex 2 (mTORC2). Phosphorylation of these specific residues is essential for Akt activation. Notably, the phosphorylation at the Thr-308 site alone helps to maintain Akt in its active form and is sufficient to activate certain downstream targets of Akt. However, enhanced substrate specificity and full activation requires an additional phosphorylation event at the Ser-473 site in the C-terminal domain [34]. Dual phosphorylation of Akt1 leads to its enhanced kinase activity that targets protein substrates, including GSK3 and Forkhead box protein O1/3a (Foxo1/3a) [35]. Structural studies have shown that phosphorylation induces conformational changes that stabilize Akt’s active form and facilitate interaction with downstream effectors involved in cell survival, proliferation, and metabolism [36].

Beyond canonical kinases, alternative phosphorylation pathways have also been identified. For instance, DNA-dependent protein kinase (DNA-PK), IκB kinase ε (IKKε), integrin-linked kinase (ILK), and mitogen-activated protein kinase-activated protein kinase-2 (MAPKAPK2) have been implicated in regulating Akt phosphorylation at Ser-473 residue under genotoxic or inflammatory stress, respectively, suggesting context-dependent flexibility in Akt regulation [37]. This adaptability allows cancer cells to maintain Akt signaling even under therapeutic or environmental pressure.

Akt has been reported to undergo simultaneous phosphorylation at Ser-477 and Thr-479 by cyclin-dependent kinase 2 (CDK2)/cyclinA2, mTORC2, and DNA-PK in response to cell cycle progression, growth factor signaling, and DNA damage [38]. These modifications are suggested to activate Akt in the nucleus and promote progression through the cell cycle. This dual Ser-477/Thr-479 phosphorylation stimulates Akt via activation loop interaction, playing a crucial role in regulating Akt activation across various physiological conditions [39].

In addition to serine and threonine, even the tyrosine residues of Akt undergo phosphorylation in response to various growth factors, including EGF, heregulin, and insulin. Specifically, EGF stimulation leads to phosphorylation of Akt at Tyr-315 and Tyr-326 by Src or protein tyrosine kinase 6 (PTK6) [40]. The Tyr-176 residue in Akt is also subject to phosphorylation by Ack1 following receptor tyrosine kinase (RTK) activation by growth factors. Phosphorylation at the Tyr-176 site is sufficient to promote Akt’s translocation to the membrane, enabling further activation through PDK1 and mTORC2. Notably, this Ack1-driven tyrosine phosphorylation of Akt persists even when PI3 kinase activity is inhibited by the PI3K inhibitor, LY294002. Tyrosine phosphorylation of Akt is required for Akt activation in response to growth factors. Site-directed mutagenesis of two tyrosine residues with phenylalanine near the activation loop of Akt completely abolishes its kinase activity [41].

Molecular dynamics analysis showed that Akt can be phosphorylated at the conserved A-loop, turn motif, and HM. In an inactive state, which happens in the absence of any external signal, the PH and kinase domains interact to form a compact Akt structure in the cytosol. However, PIK3 activation leads to phosphorylation of PIP2 to PIP3. PIP3 binds to the PH domain of Akt and recruits it to the plasma membrane, causing a change in its conformation. PIP3 recruits PDK1 to the membrane, where it phosphorylates Akt at the A-loop. mTORC2 phosphorylates Akt at the hydrophobic motif (HM) site, causing complete activation of Akt. Similarly, DNA-PK, MAPK, ILK, PKCβII, TBK1, and cyclin D1 are also involved in HM phosphorylation [42]. Irrespective of PI3K activity, Akt is co-translationally phosphorylated at the turn motif (TM) at Thr-450, Thr-451, and Thr-447 in Akt1, Akt2, and Akt3, respectively, by mTORC2. This phosphorylation is essential for AKT folding and maturation, while conferring it with stability [43]. mTORC2 phosphorylation at HM and TM sites has differential effects, as phosphorylation at the A-loop in HM increases Akt activity, whereas TM phosphorylation causes dephosphorylation of the A-loop and reduces Akt activity [44].

Ser-477 and Ser-479 in the C-terminal regulatory domain of Akt1 are phosphorylated based on cell cycle progression by cyclin-dependent kinase 2 (Cdk2/cyclinA). An association between Ser-477/Ser-479 phosphorylation and cyclin A2 expression was observed in breast cancer [45]. In Akt2, the Cdk2/cyclin A phosphorylates Ser-478 and Ser-474 in the HM site allosterically activate this protein. Amino acids 44-46 in the PH domain affect Akt phosphorylation at Ser-473 and Thr-308. For example, studies have demonstrated that the triple mutant form of Akt2 where Asp, Val, and Asp have been mutated to Gly, Pro, and Gly affects the phosphorylation of Ser-473 and Thr-308.

Akt isoforms through alternate splicing generate variants with C-termini lacking regulatory phosphorylation at HM sites but having extra C-terminal phosphorylation sites in Akt1 [46]. Upon activation, Akt translocates from the membrane to the cytoplasm and transduces signals [33]. Dephosphorylation by PTEN and INPP4B results in Akt inactivation and termination of PI3K/Akt signaling. Protein phosphatase 2A dephosphorylates the A-loop at Thr-308 in Akt1, the PH domain, and LRR protein phosphatases (PHLPP) directly dephosphorylate at HM site. Isoforms PHLPP1 dephosphorylate Akt2 and Akt3, while PHLPP2 dephosphorylates Akt1 and Akt3 [47]. In summary, phosphorylation of Akt plays a critical role in its activity and cellular localization, thereby impacting its involvement in the pathogenesis of cancers.

#### 1.5.2. Acetylation and Stability of Akt

Acetylation is a reversible PTM involving the covalent attachment of an acetyl group to lysine residues, typically mediated by histone acetyltransferases (HATs) such as p300/CBP, and removed by histone deacetylases (HDACs) [48]. While acetylation is classically associated with chromatin remodeling and transcriptional regulation, recent studies have uncovered its role in modulating the activity and localization of non-histone proteins, including Akt [49]. Acetylation is often induced under hypoxia or metabolic stress, linking PTMs to tumor microenvironment adaptation.

Akt acetylation occurs at Lys-14 and Lys-20 residues that are located within its PH domain (Table 1). Acetylation at these sites has been shown to modulate Akt’s interaction with upstream regulators to influence its association with phosphoinositides, thereby affecting its recruitment to the plasma membrane and subsequent phosphorylation by PDK1 and mTORC2 [49]. In particular, acetylation may reduce the affinity of Akt for PI (3,4,5)P_3_, attenuating its activation under certain conditions. The acetylation status of Akt is dynamically regulated by cellular stress and metabolic cues. For example, p300-mediated acetylation is enhanced under conditions of growth factor stimulation, while HDAC1/2-mediated deacetylation restores Akt’s membrane-binding capacity [50]. This suggests that acetylation serves as a modulatory checkpoint, fine-tuning Akt activation in response to extracellular signals. Furthermore, acetylation of Akt has been shown to influence its stability and subcellular localization. Acetylation at Lys-14 has been shown to enhance Akt’s stability and promote its nuclear translocation, where it can activate transcription factors involved in cell cycle progression and survival. In contrast, recent studies have shown that acetylation of lysine residues leads to the degradation of Akt. These contradictions may arise from differences in experimental models, cell types, or the presence of other regulatory factors. Future studies shall focus on developing small molecules that can promote acetylation of Akt while inhibiting deacetylases.

#### 1.5.3. Ubiquitination and Degradation of Akt

Ubiquitination has emerged as a critical non-canonical mechanism regulating Akt activation, stability, and subcellular localization [51]. Akt can undergo K63-linked ubiquitination at Lys-8 and Lys-14 residues within its PH domain. K63-linked polyubiquitination of Akt serves a scaffolding function, promoting its recruitment to the plasma membrane and facilitating phosphorylation by upstream kinases [52,53] (Table 1). In addition to K63-linked polyubiquitination, K48-linked ubiquitination has been shown to regulate AKT function and localization in cancer cells [53]. Specifically, K48-linked ubiquitination influences AKT stability and promotes its nuclear translocation, thereby altering downstream signaling pathways that contribute to tumorigenesis [53]. Notably, K48-linked ubiquitination marks the AKT for degradation by the 26S proteasome, acting as a negative regulator to turn off AKT signaling. This pathway is critical for controlling cell survival and preventing excessive, uncontrolled AKT activity, often by acting on the kinase after it has been activated. This proteosomal degradation mechanism ensures that AKT signaling is transient and reversible, preventing oncogenic, overactive kinase signaling [53]. Conversely, K63-linked polyubiquitination functions as a signaling mechanism that facilitates Akt’s recruitment to the plasma membrane. This modification is increasingly recognized as a PI3K-independent route to Akt activation, particularly in tumors exhibiting resistance to PI3K inhibitors or harboring PTEN loss [54].

The ubiquitination process is mediated by E3 ubiquitin ligases such as TNF Receptor-Associated Factor 6 (TRAF6), Neural precursor cell expressed developmentally down-regulated 4 (NEDD4-1), and S-phase kinase-associated protein 2 (Skp2), which catalyze K63-linked ubiquitin chain formation on Akt’s lysine residues, most notably Lys-8 and Lys-14 within the PH domain [55]. These modifications enhance Akt’s interaction with membrane lipids, and primes it for phosphorylation at Thr-308 and Ser-473 by PDK1 and mTORC2, respectively [56]. TRAF6 was first implicated in Akt ubiquitination in the context of EGFR signaling, where its activity was shown to be essential for Akt membrane translocation and activation [57]. NEDD4-1, on the other hand, has been linked to growth factor-induced Akt activation. Skp2-mediated ubiquitination has been associated with Akt nuclear translocation, suggesting that ubiquitination not only regulates membrane anchoring but also intracellular trafficking [58]. Ubiquitination of Akt also serves as a mechanism for regulating Akt’s degradation. Studies have identified E3 ligases that ubiquitinate Akt, leading to its proteasomal degradation. Dysregulation of this process can result in elevated Akt levels, contributing to oncogenesis. Interestingly, K63-linked ubiquitination is detected only in Akt1 and Akt2 but not in Akt3. Reasons for this selective ubiquitination of Akt1 and Akt2 but not Akt3 are yet to be discovered.

Aberrant Akt ubiquitination contributes to sustained survival signaling, metabolic reprogramming, and therapy resistance. For example, TRAF6-driven ubiquitination has been shown to promote NF-κB activation, linking Akt to inflammatory tumor microenvironments [59]. NEDD4-1 overexpression correlates with poor prognosis and enhanced glycolytic flux, underscoring the metabolic consequences of Akt ubiquitination [53]. Moreover, ubiquitination enables Akt activation in PTEN-deficient tumors, bypassing the need for PI3K-generated PIP3. This has profound implications for targeted therapy, as it renders PI3K inhibitors less effective and necessitates alternative strategies to disrupt Akt signaling. These findings highlight the diverse roles of ubiquitination in fine-tuning AKT activity and underscore its importance in cancer progression.

#### 1.5.4. SUMOylation and Localization of Akt

SUMOylation is a reversible PTM where a small ubiquitin-like modifier (SUMO) protein is covalently attached to a target protein. This process involves a cascade of enzymes, including E1, E2, and E3 ligases, and is carried out on specific lysine residues. SUMOylation affects protein stability, solubility, localization, and interactions and plays a crucial role in various cellular processes such as DNA repair, gene regulation, cell proliferation, and responses to stress [60,61]. SUMOylation of Akt typically occurs at lysine residues such as Lys-276 and Lys-301 and is catalyzed by the SUMO E2 enzyme Ubc9 and E3 ligases like the protein inhibitor of activated STAT1 (PIAS1) [62]. Rather than promoting membrane localization, SUMOylation modulates Akt nuclear functions by enhancing nuclear translocation, controlling transcription factors like FOXO and p53, and regulating transcriptional activity linked to cell survival, DNA repair, and hypoxia adaptation [62]. This modification is often induced under stress conditions such as nutrient deprivation or oxidative stress [63]. SUMOylation of Akt is also shown to affect its localization and interaction with other proteins. It is shown that SUMOylation at Lys-276 enhances Akt’s interaction with 14-3-3 proteins, influencing its subcellular localization and activity [64].

Multiple studies have confirmed that Lys-276 and Lys-301 as the key sites for SUMO conjugation on Akt [63]. Mutating Lys-276 to arginine significantly reduced Akt’s SUMOylation, indicating it is a major SUMO acceptor site. A double mutant where both Lys-276 and Lys-301 are changed to arginine resulted in an even stronger reduction in SUMOylation. The SUMOylation consensus motif around Lys-276 is conserved across different mammalian Akt isoforms, including Akt2 (Lys-277) and Akt3 (Lys-272) [64]. SUMOylation contributes to Akt’s nuclear translocation and transcriptional regulation, often intersecting with metabolic cues and stress responses. SUMOylation also has been implicated in enhancing Akt’s transcriptional activity in the nucleus, particularly under hypoxic conditions [61].

#### 1.5.5. Oxidation and Redox Regulation of Akt

Oxidation represents a non-enzymatic PTM that modulates protein function through the reversible or irreversible alteration of cysteine residues [61]. In the context of Akt signaling, oxidative modifications, particularly those induced by reactive oxygen species (ROS), have emerged as critical regulators of Akt kinase activity, subcellular localization, and downstream signaling fidelity [61,62]. Under conditions of elevated ROS, such as hypoxia, mitochondrial dysfunction, or oncogene-induced metabolic stress, the cysteine residues can undergo S-nitrosylation, S-glutathionylation, or disulfide bond formation, leading to conformational changes that impair Akt catalytic activity [61]. Although less extensively studied than phosphorylation or ubiquitination, oxidation of Akt is increasingly recognized as a key mediator of redox-sensitive oncogenic pathways.

Akt contains four key redox-sensitive cysteine residues, particularly Cys-60, Cys-77, Cys-296, and Cys-310 [61,62]. Oxidative modifications to these cysteine residues regulate Akt function through different mechanisms. Notably, Cys-60 and Cys-77 residues are located in the PH domain, which is responsible for Akt binding to phosphatidylinositol (3,4,5)-trisphosphate (PIP3) at the plasma membrane [61,62]. Reversible oxidation of Cys-60 and Cys-77 affects Akt recruitment to the plasma membrane [61]. This oxidative modification helps in fine-tuning the Akt signaling by influencing its membrane localization in response to the cellular redox status, showcasing an interplay between cysteine oxidation and phosphorylation-based signaling networks. Cys-296 and Cys-310 residues are located in the kinase domain and are crucial for the redox-mediated regulation of Akt1 [61,62]. Under oxidative stress conditions, these two cysteines can form an intramolecular disulfide bond, which inhibits Akt’s kinase activity by promoting its dephosphorylation by the protein phosphatase 2A (PP2A). Oxidation of Cys-296 and Cys-310 can also facilitate the proteasomal degradation of Akt1, indicating that maintaining the thiol status of these cysteines is important for Akt stability [61]. Mutations preventing the oxidation of these residues (C296S/C310S) protect Akt activity and promote cell survival.

Alternatively, oxidation is shown to induce Akt misfolding or sequestration in inactive complexes, effectively silencing its pro-survival signaling. Conversely, mild or transient oxidation may unexpectedly enhance Akt activity by disrupting inhibitory interactions or promoting compensatory phosphorylation [61,62]. Cysteine oxidation can also promote the degradation of Akt via the proteasome, thereby controlling the overall level of the expressed protein. Additionally, oxidative modifications interact with other PTMs, influencing Akt regulation and function. For example, oxidation of Akt reduces phosphorylation at canonical sites, targets oxidized Akt for degradation via K48-linked ubiquitination, and modulates HAT/HDAC activity, indirectly influencing Akt acetylation and transcriptional functions. These interactions suggest that oxidation is not merely a passive consequence but an active modulator of PTM crosstalk and signaling plasticity. Strategies promoting the oxidation of Akt in cancer cells are warranted to retard tumor growth; however, to date, no such selective strategies have been developed and tested. Therefore, future studies should focus on this aspect to target tumors wherein the Akt is reported to play a key role in cancer growth and development.

#### 1.5.6. O-GlcNAcylation of Akt

*O*-linked *N*-acetylglucosamine glycosylations (O-GlcNAcylation) is a dynamic and reversible PTM involving the attachment of a single N-acetylglucosamine (GlcNAc) moiety to serine or threonine residues, leading to interference with the cellular signaling and function [61,62]. Catalyzed by O-GlcNAc transferase (OGT) and removed by O-GlcNAcase (OGA), this modification serves as a nutrient sensor, integrating glucose metabolism with cellular signaling [65]. In cancer cells, O-GlcNAcylation of Akt has emerged as a critical regulatory mechanism that modulates its activity, localization, and downstream effects [66].

Akt is O-GlcNAcylated at multiple residues, including Ser-126, Ser-129, Ser-473, Thr-305, Thr-312, Thr-430 and Thr-479 [66]. The sites Thr-305 and Thr-312 are located within the Akt activation loop and are heavily involved in the crosstalk with phosphorylation, while Ser- 126 and Ser-129, sites that are identified through mass spectrometry, are also known to undergo O-GlcNAcylation and are adjacent to other phosphorylation sites. For example, Ser-473 residue is particularly notable due to its overlap with a key phosphorylation site. This competitive modification can either inhibit or enhance Akt activity depending on cellular context [66,67]. Under hyperglycemic or nutrient-rich conditions, elevated OGT activity leads to increased O-GlcNAcylation, which may stabilize Akt and promote its activation. Conversely, in some settings, O-GlcNAcylation at Ser-473 prevents phosphorylation by mTORC2, thereby attenuating Akt signaling. Moreover, studies have also identified additional O-GlcNAcylation sites in Akt at Thr-430 and Thr-479 that were reported to activate Akt by increasing Ser-473 phosphorylation [67], as well as sites at Thr-308, which were suggested to inactivate Akt by preventing phosphorylation [68]. Numerous studies have reported that the interplay between O-GlcNAcylation and phosphorylation involves an “on” or “off” effect related to mutual exclusion of these modifications on the same site. O-GlcNAcylation also occurs at Thr-305 and Thr-312 [66]. Intriguingly, O-GlcNAcylation of Akt attenuates Akt phosphorylation at Thr-308 and Akt-mediated biological functions by blocking the binding of Akt to PDK1. Although O-GlcNAcylation of Akt on Ser-126 and Ser-129 was also reported, the progress toward understanding the functional significance of O-GlcNAcylation on these two sites was hindered by the fact that Ser-126 and Ser-129 are also Akt phosphorylation sites.

The interplay between O-GlcNAcylation and phosphorylation exemplifies a reciprocal regulatory mechanism, where metabolic inputs fine-tune Akt’s signaling output. This crosstalk is particularly relevant in tumors with altered glucose metabolism, such as pancreatic, breast, and colorectal cancers. O-GlcNAcylation of Akt contributes to several hallmarks of cancer such as promoting cell growth, survival and metabolic reprogramming. This PTM is frequently elevated in cancer cells due to increased metabolic activity through the hexosamine biosynthetic pathway (HBP) [69]. O-GlcNAcylation supports the Warburg effect and enhances glycolytic flux, and elevated O-GlcNAcylation has been shown to promote anti-apoptotic signaling and cell cycle progression, especially under stress conditions.

**Table 1:** List of post-translational modifications of Akt and their functional/oncogenic implications.

Type of Modification	Key Residues Modified	Functional and Oncogenic Implications	Reference
Phosphorylation (Ser/Thr)	Thr308, Ser473, Thr305, Thr309, Ser472/474	Core activation mechanism of Akt; dual phosphorylation enables full kinase activity, substrate recognition, and pro-survival signaling; drives proliferation, metabolism, and therapy resistance	[70]
Phosphorylation (Ser/Thr, non-canonical sites)	Ser477, Thr479, Thr450/451/447	Context-dependent activation linked to cell cycle progression, DNA damage responses, and protein stability; regulates nuclear Akt signaling	[71]
Tyrosine phosphorylation	Tyr176, Tyr315, Tyr326	Enables PI3K-independent membrane recruitment and activation of Akt; sustains growth-factor signaling	[72]
Acetylation	Lys14, Lys20	Modulates PH domain binding to phosphoinositides, Akt stability, and nuclear localization; integrates metabolic and stress signals	[73]
Ubiquitination	Lys8, Lys14	Promotes Akt membrane recruitment and phosphorylation; supports oncogenic signaling independent of PI3K	[50]
Ubiquitination (K48-linked)	Multiple lysines	Targets Akt for proteasomal degradation; limits signal duration; dysregulation leads to sustained Akt signaling in cancer	[53]

## 2. Variations in the Cellular Localization Contributes to Differential Activity of Akt in Tumor Cells

Localization of Akt plays an important role in its activation and the function of a cell. Akt is a cytosolic protein [74]. Activation of Akt is a multistep process involving translocation to the cell membrane followed by the phosphorylation of Thr-308 and Ser-473 [75]. Translocation of Akt is mediated by the PH domain, which has a very high affinity for the 3′-phosphorylated phosphoinositides 3,4,5-trisphosphate (PI-3,4,5-P_3_) and PI-3,4,-P_2_. These PI-phosphates are produced by PI3K [76]. Phospholipid binding triggers the translocation of AKT kinases to the plasma membrane, wherein AKT is phosphorylated at Thr-308/309 in the kinase activation loop and Ser-473/474 in the carboxyl-terminal tail [77]. In summary, the translocation of Akt from the cytosol to the cell membrane is a key step in the complete activation of Akt.

Interestingly, recent studies have demonstrated the key role of nuclear Akt in the maintenance of stem-like cells in breast cancers [78]. Authors of this study have shown that overexpression of Akt with nuclear localization signal (NLS) could increase the percentage stem-like cells in SKBR and MDA-MB-468 cell lines. Mechanistically, breast cancer cells overexpressing NLS-Akt downregulate p21Waf1/Cip1, thereby preventing the accumulation of cells in the G0/G1 stage of the cell cycle [79]. As a result, these stem-like cells begin to proliferate and form colonies, thereby promoting drug resistance and enhancing metastatic spread of tumor cells [80]. In summary, NLS-Akt expression is an important event in the transformation of breast cancer cells into stem-like cells [78]. Hence, approaches preventing this translocation step play a vital role in developing potent inhibitors of Akt. However, since Akt is devoid of NLS, it is not clearly known how Akt is transported to the nucleus. Hence, studies are warranted to find the pathways that regulate Akt nuclear localization. Moreover, studies should also focus on the involvement of carrier proteins that facilitates the nuclear localization of Akt. Furthermore, mounting evidence has mentioned that isoform-specific nuclear localization of Akt plays a role in its function. For instance, a study by Wainstein E. et al., 2022, has shown that among different isoforms of Akt, Akt2 is localized to the nuclear region, but Akt1 and Akt3 are not [81]. Studies have shown that Akt1 is localized in cytosol, but Akt3 resides at the nuclear envelope or in the vicinity of mitochondrial membranes. In summary, cellular localization of Akt plays an important role in exhibiting its activity. Future studies should focus more on developing compounds that effectively target the cellular localization of Akt.

## 3. Genetic Alterations of Akt Isoforms and Their Role in the Tumorigenesis

To date no mutations in the catalytic domain of Akt isoforms have been reported in cancers. However, a somatic mutation involving Glu-17 to Lys (E17K) in the PH domain of *Akt1* gene has been reported and found to cause conformational change, membrane localization and weak constitutive activation of Akt1 [82]. The rate of the Akt1 E17K mutation in breast cancers is about 6.3%. *Akt1 E17K* oncogene with additional mutations has been shown to promote tumorigenesis in some breast cancers. Patient-derived xenograft (PDX) studies have demonstrated that *Akt1 E17K* mutation is confined in hormone receptor positive (HR+) luminal breast cancers [83]. The mutations in Akt2 and Akt3 are rare. Genetic alteration in *Akt1* is higher in metastatic breast cancer compared to primary breast cancer, and hence only Akt1 is considered as an actionable target [84]. However, Akt2 is expressed in 3% of breast cancers, while Akt3 is expressed in triple negative breast cancers (TNBC) [85]. Interestingly, studies have shown that the mRNA expression of Akt1 correlates with poor prognosis of the basal-like (BL2) subset of TNBC [86].

Although Akt contributes to tumor growth by increasing proliferation or decreasing cell death while enhancing metastasis from the primary to a secondary site, the isoform-specific functions are yet to be elucidated. For instance, a study has shown that Akt1 and Akt2 have contradictory roles in initiation and tumor progression. In several breast cancer cells, overexpressed Akt2 enhanced invasion through collagen IV matrix and increased metastasis in MDA-MB-435 xenografts [87]. In a transgenic mouse model, activated Akt1 accelerated ErbB2-induced tumorigenesis by enhancing cyclin D1 and cell proliferation but suppressed lung metastases [87]. However, activated Akt2 did not affect tumor development but caused lung metastases.

Further studies have also shown that Akt isoforms play distinct roles in the development and progression of breast cancer. For example, a study has shown that Akt1 is associated with the majority of the oncogenic functions. Recent studies have also demonstrated that Akt1 and Akt2 have opposing roles, as Akt1 promotes tumor initiation by enhancing cell proliferation, cell survival and tumor growth, but inhibits tumor progression, whereas Akt2 aids in the progression of tumors by increasing migration, invasion and metastasis and is associated with worse clinical outcomes and considered as a useful target for breast cancer [88]. Akt3 expression was previously restricted to neuronal cells but is now known to play a critical role in TNBC. However, a consensus regarding isoform-specific functions of Akt is not possible in any particular subtype of breast cancer. Akt1 knockdown in breast cancer cells led to increased migration and invasion, whereas its genetic ablation in mouse models decreased tumor metastasis [89]. Hyperactivation of Akt1 decreased metastasis in vitro and in vivo models [90]. Similarly, the role of Akt isoforms in migration, invasion, and metastasis is reported, but their involvement in cell proliferation and tumorigenesis requires further studies. For instance, Akt2 has been shown to decrease, or increase, or have no effect on cell proliferation and tumor growth [91]. Therefore, studies are immediately needed to delineate the isoform-specific functions of Akt in the tumor initiation, progression and metastatic spread.

## 4. Small Molecule Inhibitors of Akt in the Treatment of Cancers

Since several in vitro and in vivo studies have shown the beneficial effects of targeting Akt, efforts have been made to identify small molecule inhibitors that can reduce the Akt expression and/or activity in cancer cells. In this section of the review, we will be discussing the recent advancements in the development of Akt-specific inhibitors.

Numerous small molecules exhibiting potent Akt inhibitory activity have been identified through both in vitro and in vivo experimental approaches [92]. However, those taken further for clinical evaluation are scarce. Very few inhibitors such as the Capivasertib (AZD5363) and Ipatasertib (GDC-0068), which are ATP-competitive inhibitors, have been tested in phase I and II clinical trials for both mono- and combination therapeutic regimens. However, their success in inhibiting cancer cells’ growth is marginal. Lack of clinical success with Akt inhibitors is primarily due to the presence of isoforms, induction of hyperglycaemia (metabolic toxicity) and off-target actions. Furthermore, studies have reported re-wiring of pathway networks and feedback activation of signaling cascades. As a result, the tumors tend to exhibit resistance, leading to the failure of Akt inhibitors in clinic. For example, studies have demonstrated the activation of receptor tyrosine kinases such as EGFR and IGF-1R upon the inhibition of Akt. Similarly, activation of isoforms other than the one inhibited and mTOR have also been reported in the drug-resistant tumor cells. Since the ATP-binding pocket of Akt is highly conserved among kinases, it is a challenge to develop a specific Akt inhibitor. Therefore, the discovery of allosteric inhibitors, which are highly Akt-specific and isoform-selective, is promising. For example, MK-2206 and Miransertib (ARQ092) are allosteric pan-Akt inhibitors with marked specificity and lesser toxicity and side-effects. Though MK-2206 has been extensively investigated in breast cancer clinical studies, its expected efficacy was not observed. Miransertib and its next generation inhibitor Arq751 are in clinical studies and are effective in targeting Akt^E17K^. However, none of these have reached phase III in clinical trials. Hence, the search for potent Akt inhibitors that show clinical success still continues.

### 4.1. Covalent Inhibitors of Akt

Covalent-allosteric compounds have been investigated for Akt inhibition. These inhibitors have shown high selectivity with irreversible covalent modification of noncatalytic cysteines in Akt. For instance, Borussertib, a covalent-allosteric inhibitor, showed better efficacy in comparison to other Akt inhibitors. The compound had sustained target residence time and significant activity in cancer cells with altered PI3K/Akt signaling. It showed in vivo efficacy in combination treatment with the MEK-inhibitor Trametinib in KRAS mutant PDX models [93]. However, this compound suffers from poor pharmacokinetic properties, which limit its oral application. Future studies should consider modifying the structure with a structure-based drug design approach to improve its pharmacokinetic properties.

### 4.2. Synthetic Inhibitors Targeting Akt in Cancers

#### ATP-Competitive Inhibitors

Several ATP-competitive Akt inhibitors have been synthesized and examined. The first synthetic and selective inhibitor of Akt, i.e., NL-71-101, was developed from a combinatorial library screening. NL-71-101 was synthesized by modifying the protein kinase A inhibitor H-89. NL-71-101 is a potent inhibitor of Akt activity in Caco-2 cells. This discovery highlighted the role of Akt in Caco-2 cell function and suggested that targeting Akt could be a viable strategy for cancer treatment, paving the way for the development of Akt inhibitors [94]. H-89 has 70-fold the protein kinase A inhibitory activity in comparison to Akt. However, the derivative NL-71-101 exhibits 2–3-fold more Akt inhibitory activity than protein kinase A inhibition, as observed by its IC50 of 3.7 μM and 9.0 μM against Akt and PKA, respectively [95]. NL-71-101 induced apoptosis in OVCAR-3 ovarian carcinoma cells at high concentrations (>25 μM). The isoquinoline functional group, sulfonamide moiety, and the diamine linker are responsible for the Akt1 activity of H-89 as modification of its structure results in loss of activity. A selective analog of H-89 with higher Akt inhibitory activity has been synthesized with an IC50 of 0.17 μM [96]. But, there is no evidence that this molecule is progressed further into formal preclinical development or clinical trials.

Azepane derivatives with Akt inhibitory activity have been developed from the fungal metabolite Balanol. Balanol ((4R)-4-(2-fluoro-6-hydroxy-3-methoxy-benzoyl)-benzoic acid (3R)-3-[(pyridine-4-carbonyl)amino]-azepan-4-yl ester) is a potent PKA inhibitor with an IC50 of 5.0 nM but is highly plasma unstable, limiting its application as a drug. However, by replacing the ester moiety in its structure with an amide group, Azepane inhibitors of Akt were obtained. An Azepane derivative, N-[(3R,4R)-4-[4-(2-fluoro-6-hydroxy-3-methoxy-benzoyl)-benzylamino]-azepan-3-yl]-isonicotinamide, was highly stable and exhibited potent Akt inhibitory activity with an IC50 of 4.0 nM [97].

Lead optimization has led to the discovery of Aminofurazan derivatives as pan-Akt kinase inhibitors. One such ATP-competitive inhibitor is the Aminofurzan GSK690693 with the ability to inhibit all three isoforms of Akt. It has IC50 values of 2.0, 13.0, and 9.0 nM against Akt1, 2, and 3, respectively [98]. X-ray cocrystal structure has shown that the compound binds to the kinase domain of Akt2 at the ATP binding pocket. It inhibited Akt activity and reduced the phosphorylation of GSK3β in immunocompromised mice and reduced tumor growth in BT474 xenografts. It inhibited the proliferation of hematologic neoplasia and acute lymphoblastic leukemia cell lines [99]. However, due to its ability to induce transient hyperglycemia, further clinical development was terminated.

The heterocyclic 6-5 fused ring, 7-azaindole, was identified as an important fragment in Akt inhibitors. Fragment-based screening and scaffold optimization led to the discovery of 6-phenylpurines as potent Akt inhibitors [100]. This lead compound was used as a scaffold and further developed as pyrrolo[2,3-*d*]pyrimidine derivative. Such fragment-based screening and protein–ligand crystallography led to the identification of potent and highly selective Akt inhibitors 6-(piperidin-1-yl) purine, 4-(piperidin-1-yl)-7-azaindole, and 4-(piperidin-1-yl) pyrrolo[2,3-*d*]pyrimidine. These 4-aminopiperidine derivatives at nanomolar concentrations inhibited Akt2 and exhibited antiproliferative activity. 4-aminomethylpiperidine and 4-aminopiperidine showed different binding conformations. The coupling of the 4-aminopiperidine and pyrrolopyrimidine moieties provided more selectivity for Akt [101].

CCT128930, an advanced lead pyrrolopyrimidine Akt inhibitor, has also been discovered using fragment- and structure-based screening. It exhibits selectivity for Akt over PKA due to the difference in a single amino acid in its structure [102]. It prevents phosphorylation of Akt substrates and exhibits antiproliferative activity in multiple tumor cell lines and U-87 MG and HER2-positive, PIK3CA-mutant BT474 human breast cancer xenografts. It caused G(1) arrest in PTEN-null U-87 MG by blocking AKT pathway. In U-87 MG tumor xenografts, CCT128930 blocked the phosphorylation of downstream AKT biomarkers. The study also led to the development of a novel biomarker assay for screening of Akt inhibitors using human hair follicles, as it contains Akt substrate PRAS40.

Pyrrolopyrimidine scaffold served as another source of Akt inhibitors [103]. For example, potent Akt inhibitors have been developed by substituting piperidine ring in CCT128930, which resulted in 3-aminopyrrolidines with preferred selectivity for PKA [102]. A pyrrolopyrimidine with an anilinotriazole structure has shown potent Akt1 inhibitory activity. The substitution of anilinotriazole with an imidazopiperidine led to the discovery of the spiroindolines scaffold as linker to the pyrrolo[2,3-*d*]pyrimidine [104]. The metabolism and bioavailability of CCT128930 was improved by modifying the 4-amino-4-benzylpiperidine moiety to 4-amino-piperidine-4-carboxamide, resulting in AZD5363 development [101]. AZD5363 inhibits all Akt isoforms with a potency of ≤10 nmol/L and has good pharmacokinetics properties. It inhibited the proliferation of 41 solid and hematological tumor cell lines at ≤3 μmol/L. It has highest sensitivity in breast cancer cells and increased the activity of the chemotherapeutic agents docetaxel, lapatinib and trastuzumab in breast cancer xenografts. Similarly, in the presence of pan-erbB tyrosine kinase, it showed improved activity on (HER2)-amplified breast cancer [105]. AZD5363 is in phase I and II clinical trials for breast, gastric and prostate cancers [106].

Optimization of 6,7-dihydro-5H-cyclopenta[d]pyrimidine compounds led to the discovery of Ipatasertib (GDC-0068, RG7440). It is a potent inhibitor of all Akt isoforms with no marked activity against PKA [107]. Ipatasertib inhibits Akt signaling in cancer cell lines and in tumor xenograft models by blocking cell cycle progression [108]. Ipatasertib is orally bioavailable and in clinical phase I and II trials (NCT02430363 and NCT02301988). It is well tolerated in combination with Paclitaxel, and hence the efficacy of this combination is being evaluated in metastatic TNBC clinical trials [109].

Similarly, the potency of the Akt inhibitors A-674563 and A-443654 has been improved by structural modification. An isoquinolone ring was developed by adding an indole ring to the aliphatic side chain and constraining rotatable bonds in the pyridine rings. A-443654T is an isoquinoline with a transformed indazole ring, while A-674563 has the indole replaced with a phenyl moiety, resulting in increased oral bioavailability [110]. Both are potent ATP-competitive inhibitors of Akt, with A-443654 having equal potency against Akt isoforms and 40-fold higher selectivity for Akt over PKA [111]. A-443654 has higher potency than A-674563 but has similar in vivo antitumor activity and increases the efficacy of Paclitaxel in prostate carcinoma xenografts. Similar heterocyclic 6-5 fused rings have been developed as potent Akt inhibitors, viz., Azaindazole and 4,7-diazaindazole. Studies have shown that pan-Akt inhibitors are made up of Dihydrothieno- and dihydrofuro-pyrimidine scaffolds. Others are the 1*H*-indazole-4,7-diones series with dual inhibitory effects on Akt1 activity and phosphorylation in the PC-3 and GSK690693 aminofurazane derivative developed from imidazo[4,5-*c*]pyridine core [112,113,114].

AT7867 is a phenylpyrazole derivative with pan-Akt inhibitory potential. It was developed using fragment- and structure-based drug design, and its ability to bind in the ATP binding site was confirmed by X-ray crystallography studies [115]. In vitro studies have demonstrated that AT7867 inhibits Akt isoforms, and the downstream p70 S6 kinase thereby suppresses cell proliferation and induces the apoptosis in various cancer cell lines [116]. AT13148 is another derivative developed from the 4-phenylpyrazole scaffold of AT7867 and hence has similar structural and functional profiles. It is a multi-AGC kinase inhibitor of Akt, p70 S6K, PKA, ROCK, and serum and glucocorticoid-inducible kinase with the ability to trigger apoptosis in cancer cells.

Thiophenecarboxamides and derivatives are potent Akt inhibitors, with their structure playing a key role in lead optimization. The amide bond and the optimal length of two carbon atoms between the phenyl ring and the amide nitrogen are important parts of their structure [117]. 2-pyrimidyl-5-amidothiophene is an Akt3 ATP-competitive inhibitor. The replacement of the pyrimidine moiety with a pyrazole ring led to synthesis of Afuresertib (GSK2110183), and thiophene in thiazole led to DC120 [118,119]. DC120 has anti-proliferative activity in CNE2 and MDA-MB-453 cell lines and inhibited CNE2 tumor in xenograft studies. It mediates apoptosis by elevating the expression of cleaved caspase-3 while reducing the phosphorylation of FKHR, glycogen synthase kinase 3β (GSK-3β) and mTOR in a dose- and time-dependent manner [120].

Afuresertib (GSK2110183) has subnanomolar potency against Akt1 in comparison to Akt2 and Akt3. Its in vitro efficacy was confirmed in acute lymphoblastic leukemia, non-Hodgkin’s lymphoma, and chronic lymphocytic leukemia, and it showed clinical efficacy in hematological malignancies, especially multiple myeloma. In phase I/II clinical trials for relapsed or refractory myeloma, the compound in combination with Bortezomib and Dexamethasone showed a 41% response rate [119,121,122]. Thus, Afuresertib is safe and well tolerated and has low incidence of hyperglycemia with the improved kinase selectivity. Uprosertib (GSK2141795) is a structural analog of Afuresertib, with a bioisostere furan ring instead of the thiophene core, and has higher Akt inhibitory potency. Clinically, it has been tested alone or in combination with Trametinib against various cancer types [119].

The major limitation with ATP-competitive inhibitors is low selectivity against protein kinases and among the Akt isozymes, mainly due to similarity in catalytic domain. Screening of compounds for Akt-specific and isoform-selective inhibitors led to the discovery of allosteric Akt inhibitors [123]. The allosteric inhibitors target active sites, have greater specificity, low side-effects and low toxicity [124].

### 4.3. Allosteric Inhibitors of Akt

The first allosteric Akt inhibitor is a 2,3-Diphenylquinoxaline derivative, which had potent Akt1 inhibitory activity, but not on the mutated Akt enzyme lacking the PH domain [125]. It was not only a pan-Akt inhibitor but also showed selectivity over PKA, PKC and SGK [126]. Its derivative 5,6-diphenyl-pyrazin-2(1*H*)-one scaffold had Akt1 and Akt2 inhibitory activity. Imidazoquinoxaline has been used as template for developing allosteric Akt inhibitors. For instance, replacement of core 1,4-quinoxaline with a naphthyridine improved the compound’s features including basicity, polarity and the solubility and significant Akt2 inhibition [123,126,127]. Another example is orally active Akt inhibitor MK-2206, a triazolo[3,4-*f*][1,6]naphthyridin-3(2*H*)-one derivative. The compound binds at the interface of the kinase domain and the PH domain, locking it in a closed conformation, thus blocking the active state. The compound is a highly potent pan-Akt inhibitor which reduces phosphorylation of p-Akt Thr-308 and p-Akt Ser-473 and downstream targets, GSK-3β, PRAS40, FoxO1/FoxO3a, and Bad [128,129]. It induces dose-dependent G1-phase cell cycle arrest and apoptosis with CYP3A4-mediated oxidation [130,131]. Preclinical studies showed its efficacy in combination with Doxorubicin, Camptothecin, Gemcitabine and 5-fluorouracil (5-FU), Carboplatin, Erlotinib and Lapatinib in NCI-H460 and A2780 cells lines. In phase I studies, it was tested alone and in combination with Trastuzumab or Lapatinib in different breast cancer types [132]. However, in clinical trials for acute myelogenous leukemia (AML) and in advanced colorectal cancer, MK-2206 alone or in combination with other drugs, it did not yield the desired efficacy [133,134].

#### 4.3.1. Alkylphospholipids (ALPs)

Alkylphospholipids (ALPs), developed from lysophosphatidylcholine, are long hydrocarbon chain-containing molecules that can easily partition and accumulate in the plasma membrane [135]. ALPs can induce apoptosis, but can also cause non-apoptotic cell death [136]. ALPs avert membrane recruitment of the PH domain of Akt by either disrupting microdomains and/or displacing the ligands. Thus, Akt failed to attain a conformation, which is required for phosphorylation and activation [137]. Edelfosine (Et-18-OCH_3_), the first synthetic ALP analog, has shown anti-proliferative effects, with decreased p-Akt Ser473 levels in treated cells and reduced phosphorylation of mTOR [138,139]. However, it has high toxicity and low selectivity, limiting its clinical application [140]. Its thioether analog, Ilmofosine (BM 41.440), has shown antitumor activity in solid tumor cells, but its gastrointestinal toxicity is dose-limiting [141]. As the glycerol moiety in ALP was not required for anti-tumor activity, it was replaced with phosphoester chain for developing next generation inhibitors. The hexadecylphosphocholine compound known as Miltefosine has been tested in clinical studies as an oral therapy for soft tissue sarcomas. Advanced CRCs showed high hemolytic toxicity on intravenous application [142,143]. Hence, it was used alone or in conjunction with other therapies for cutaneous breast cancer and found to be effective and tolerable as a topical application agent [144]. Replacing choline moiety with a piperidine in Miltefosine led to the development of a stable and GIT-tolerant compound D-21266 (Perifosine). Perifosine has been tested in several clinical trials against different tumors but so far has been effective in sarcoma and Waldenstrom macroglobulinemia. Clinical trials are ongoing for Perifosine in combination with Capecitabine for CRC in combination with Bortezomib and other drugs for multiple myeloma [145]. A homolog of Miltefosine, Erucylphosphocholine (ErPC), was developed, but it had poor solubility. Hence, a structural analog with improved solubility was screened and identified: Erufosine (ErPC3, erucylphosphohomocholine). Both compounds exhibited better BB barrier properties and hence are suitable in glioblastoma treatment [146,147]. Preclinical studies have shown that these compounds have additive effects in radiation therapy [148].

#### 4.3.2. Indole-3-Carbinol (I3C) and Analogs

I3C and its analogs have been investigated for Akt inhibitory activity to develop stable compounds. For example, 3-chloroacetylindole is an ATP non-competitive Akt1 and Akt2 inhibitor, which suppresses growth and induces apoptosis [149]. Using diindolylmethane as a template, many indole analogs were developed, and among these, indolo[2,3-b]carbazole derivative was promising. It exerted potent oral anticancer activity with no toxic effects in experimental animals [150]. Preclinical pharmacokinetic studies showed low bioavailability due to its poor solubility and high membrane permeability [151]. OSU-A9, an indole-3-carbinol derivative, had good stability and inhibited Akt signaling in MCF-7 cells by dephosphorylation of Akt and downstream substrates GSK-3β and IKKα [152].

#### 4.3.3. PH-316, a Sulfadiazine Derivative

PH-316, a sulfadiazine derivative, was developed by computational screening to determine its ability to bind PH domain in Akt1. Although it inhibited Akt and cell proliferation in HT-29 cell lines, in preclinical models, the blood concentration required for Akt inhibition was not achieved due to its rapid metabolism of the azo bond [153]. Structural modifications of PH-316 led to PHT-427 (4-dodecyl-*N*-(1,3,4-thiadiazol-2-yl) benzenesulfonamide). PHT-427 inhibits Akt with an IC50 of 6.3±0.9 μmol/L in Panc-1 cells and induced apoptosis at 20 μmol/L [92]. It inhibited tumor growth in PDX models, and in combination with Paclitaxel, Gemcitabine, and Erlotinib, showed synergistic activity in a Panc-1 tumors, non-small-cell lung cancers and breast cancer [154].

Based on fragment synthesis using Akt1, inhibitors of PIP3/PH domain binding known as PIT-1 and PIT-2 were developed. These compounds inhibited binding of PIP3 to the PH domains of PDK1, GRP1 and ARNO [155]. To improve the solubility of PIT-1, its dimethyl analog, DM-PIT-1, was developed. SAR studies have shown that Thiourea and hydroxyl groups in these compounds play a critical role in exhibiting the anticancer activity [156]. Pyrazole derivatives N-[(1-methyl-1*H*-pyrazol-4-yl) carbonyl]-N′-(3-bromophenyl)-thiourea are another set of potent apoptosis inducing agents with Akt inhibitory activity [157]. In vitro and in vivo studies evaluating these compounds are warranted to evaluate their safety and efficacy in clinical trials.

Purine derivative, API-2 [(Triciribine (TCN)], is a potent Akt inhibitor which inhibits cell growth and induces apoptosis [158]. Intracellularly, API-2 is metabolically activated by adenosine kinase to its monophosphate analog TCN-P [159], which binds to the PH domain in PIP3 binding pocket and thereby disrupts the phosphorylation of Akt. Plasma membrane recruitment of Akt via PIP3 is prevented by competitive binding to the PH domain or by inducing conformational changes preventing PIP3 activation [160]. TCN at 10 μM inhibits Akt phosphorylation and its downstream signaling, resulting in cell cycle arrest and apoptosis in T cell acute lymphocytic leukemia cells [161]. TCN is also reported to induce apoptosis in prostate cancer cells [162]. In clinical trials against solid neoplasms and hematological malignancies, TCN and TCN-P showed toxicity. Hence, a preferable way to use these molecules is by combination therapy [163]. Since toxicity was observed with the purine analogs TCN and TCN-P, an attempt was made to develop Akt inhibitors from pyrimidines. The most potent orally bioavailable pyrimidine-based Akt inhibitor is the N-(4-(5-(3-acetamidophenyl)-2-(2-aminopyridin-3-yl)-3H-imidazo[4,5-b]pyridin-3-yl)benzyl)-3-fluorobenzamide [164].

Next, Akt inhibitors with negative regulatory function on binding to hydrophobic clusters in the ATP-binding cleft have been developed. ARQ 092 with a core structure of 3-(3-phenyl-3H-imidazo[4,5-b]pyridin-2-yl)pyridin-2-amines inhibits all three isoforms by binding to inactive and unphosphorylated Akt at subnanomolar affinity. It inhibited AN3CA growth in xenograft models on oral administration [165]. Along with its congener, ARQ 751, potent activity was seen in leukemia, breast, endometrial and CRC cell lines [166].

### 4.4. Irreversible Inhibitors

Covalent inactivators of Akt have been developed using derivatives of phenylalanine vinyl ketone. One such compound showed Akt inhibition by binding to Cys310 and suppressed HCT116 and H460 cell growth [167]. Compounds derived from 1,6-naphthyridinone and imidazo-1,2-pyridine templates target Cys296 or Cys310, are highly selective towards Akt, and can target all three isoforms [168].

## 5. Inhibitors of Akt from Natural Sources

Natural products and their derivatives are amenable to large-scale synthesis and can be easily conjugated to other molecules for targeted delivery. Structural modification also helps to improve the pharmacological properties of these natural compounds. Certain natural products occur in the diet and are consumed regularly and hence presumed safe. Therefore, natural products can be explored for the discovery of anti-cancer drugs.

### 5.1. Plant Derived Small Molecule Inhibitors of Akt

Indole-3-carbinol, a natural compound from *Brassica* species, has been shown to inactivate Akt, thereby inhibiting tumors in rodents [169]. The compound has low stability and hence is converted to 3,3′-diindolylmethane under acidic conditions [170].

Tetracyclic triterpenoids, isolated from the oleogum resin of Boswellia carterii, has demonstrated potent Akt inhibitory activity. 3-oxo-tirucallic acid is a weak inhibitor of Akt, but the other derivative 3α- and 3β-acetoxy-tirucallic acid effectively block Akt1 activity at concentrations as low as 1 μmol/L. Acetoxy-tirucallic acids inhibit phosphorylation of GSK-3β and reduce β-catenin and c-Myc levels in PC-3 cell line. Mechanistic studies have shown that the Tirucallic acid binds to the PH domain of Akt. In mice, administration of 3β-acetoxy-tirucallic at 10 μmol/kg significantly reduced xenotransplanted PC-3 tumors in 2 weeks [171]. Further studies evaluating this compound in higher animals, and PDX are warranted to consider this molecule for clinical usage.

Apigenin, a flavonoid abundant in fruits, vegetables and beverages shows anticancer activity by the inhibition of PI3K/Akt/mTOR signaling, either by direct downregulation of PI3K/Akt activity or by indirect activation of AMPK-TSC axis [172].

Curcumin (diferuloylmethane), a polyphenol isolated from Curcuma longa, primarily targets Akt/mTOR signaling and thus shows inhibitory activity in many cancer cell lines. It inhibits phosphorylation of S6K1 and 4E-BP1, which are the downstream effectors of mTORC1 at <40 μM. Additional studies have shown that Curcumin can also inhibits phosphorylation of mTORC2 substrate Akt at >40 μM. Disruption of mTOR-raptor complex and activation of protein phosphatase 2A inhibit Akt/mTOR signaling [173].

Fisetin, (3,3′,4′,7-tetrahydroxyflavone) is a plant-derived flavonol enriched by common dietary sources such as strawberries, apples, persimmons, grapes, and onions and has been implicated as an inhibitor of oncogenic PI3K–Akt signaling [174]. Mechanistically, fisetin suppresses Akt pathway output by inhibiting upstream PI3K activity and reducing phosphorylation/activation of Akt with downstream attenuation of mTOR, thereby shifting the balance toward growth arrest and apoptosis in multiple tumor models [175]. Fisetin has been reported to function as a “dual” pathway inhibitor (PI3K/Akt and mTOR), providing a rationale for its anti-proliferative and pro-apoptotic effects and supporting further evaluation as a lead scaffold or adjuvant strategy targeting Akt-axis dependency [176].

Indole-3-carbinol (I3C), an indole phytochemical derived from the hydrolysis of glucobrassicin in cruciferous vegetables such as broccoli, cabbage, cauliflower, and Brussels sprouts, is well recognized for its inhibitory effects on oncogenic Akt signaling [177]. Studies demonstrate that I3C suppresses the PI3K/Akt pathway by reducing Akt phosphorylation and activity, leading to downstream inhibition of mTOR signaling, altered cell-cycle regulator expression, and induction of apoptosis [178]. In cancer models characterized by constitutive Akt activation, including breast, prostate, and cervical cancers, I3C has been shown to promote G1 cell cycle arrest through modulation of cyclin-dependent kinases and enhancement of tumor suppressor pathways while simultaneously impairing survival signaling mediated by Akt [179]. I3C and its in vivo condensation product 3,3′-diindolylmethane (DIM) exert complementary effects on Akt-dependent transcriptional programs, reinforcing their potential utility as dietary-derived Akt pathway inhibitors with chemopreventive and therapeutic relevance [180].

Resveratrol (3,5,4′-trihydroxystilbene) is a naturally occurring polyphenolic stilbene predominantly found in grapes, berries, peanuts, and red wine and is widely recognized for its ability to inhibit Akt-driven oncogenic signaling [181]. Mechanistic studies demonstrate that resveratrol suppresses the PI3K/Akt pathway by reducing Akt phosphorylation and kinase activity, leading to downstream inhibition of mTOR signaling and attenuation of survival, proliferation, and metabolic programs in cancer cells [182]. This Akt inhibition is often accompanied by activation of tumor suppressive pathways, including AMPK and p53, as well as suppression of NF-κB–dependent transcription, thereby promoting apoptosis and cell-cycle arrest [183]. Resveratrol has been shown to sensitize tumor cells to chemotherapy and radiotherapy, underscoring its relevance as a plant-derived Akt pathway modulator with both chemopreventive and therapeutic potential [184].

Caffeine (1,3,7-trimethylxanthine), a naturally occurring methylxanthine alkaloid abundantly present in coffee, tea, cocoa, and several medicinal plants, has been reported to negatively regulate Akt-mediated survival signaling in cancer cells [185]. Caffeine interferes with the PI3K/Akt pathway primarily by suppressing Akt phosphorylation and downstream mTOR signaling, leading to reduced cellular proliferation and enhanced apoptosis. In several cancer models, caffeine-mediated Akt inhibition is associated with activation of DNA damage response pathways (ATM/ATR), cell cycle checkpoint enforcement, and sensitization of tumor cells to chemotherapeutic agents and ionizing radiation [186]. Additionally, caffeine has been shown to modulate upstream regulators of Akt, including PI3K and growth factor receptor signaling, thereby attenuating pro-survival and anti-apoptotic signaling cascades [187]. These findings support caffeine as a plant-derived bioactive compound with context-dependent Akt inhibitory activity and potential value in Akt-driven malignancies.

Epigallocatechin gallate (EGCG) is a biologically active catechin found in green tea (Camellia sinensis) and is extensively studied for its inhibitory effects on Akt-driven oncogenic signaling [188]. EGCG suppresses the PI3K/Akt pathway by directly or indirectly inhibiting PI3K activity, resulting in reduced Akt phosphorylation and downstream attenuation of mTOR and its effector pathways, including protein synthesis and cell survival signaling [189]. EGCG-mediated Akt inhibition is frequently accompanied by modulation of upstream receptor tyrosine kinases), activation of AMPK, and suppression of NF-κB–dependent transcription, thereby promoting apoptosis and cell cycle arrest in diverse cancer models [190]. In malignancies characterized by constitutive Akt activation, such as breast, prostate, colorectal, and cervical cancers, EGCG has demonstrated chemopreventive efficacy and the ability to sensitize tumor cells to conventional therapies, supporting its relevance as a plant-derived Akt pathway inhibitor with translational potential [191].

Celastrol is a bioactive pentacyclic triterpenoid quinone methide isolated from the medicinal plant *Tripterygium wilfordii* and is recognized as a potent natural inhibitor of Akt-mediated oncogenic signaling. Celastrol suppresses the PI3K/Akt pathway by inhibiting Akt phosphorylation and kinase activity, leading to downstream attenuation of mTOR signaling and disruption of pro-survival and anti-apoptotic pathways [192]. Akt inhibition by celastrol is frequently coupled with induction of oxidative stress, activation of stress-responsive kinases, suppression of NF-κB signaling, and modulation of heat shock protein 90 (HSP90), a critical chaperone for Akt stability [193].

Butein (3,4,2′,4′-tetrahydroxychalcone), a naturally occurring polyphenolic chalcone isolated from several medicinal plants, including *Rhus verniciflua*, *Dalbergia odorifera*, and *Semecarpus anacardium*, has been identified as a potent inhibitor of Akt-mediated oncogenic signaling [194]. Butein suppresses the PI3K/Akt pathway by inhibiting Akt phosphorylation and kinase activity, resulting in downstream attenuation of mTOR signaling and reduced activation of survival and proliferative effectors [195]. Akt inhibition by butein is frequently accompanied by suppression of NF-κB signaling, modulation of STAT3 activity, and induction of mitochondrial-dependent apoptosis [196]. Butein has been shown to induce cell cycle arrest, promote apoptotic cell death, and enhance sensitivity to chemotherapeutic agents, supporting its relevance as a plant-derived Akt pathway inhibitor with significant anticancer and chemosensitizing potential [197].

Capsaicin (8-methyl-N-vanillyl-6-nonenamide) is a bioactive vanilloid alkaloid derived from *Capsicum* species. It has been extensively investigated for its inhibitory effects on Akt-driven oncogenic signaling [198]. Capsaicin suppresses the PI3K/Akt pathway by reducing Akt phosphorylation and kinase activity, leading to downstream inhibition of mTOR signaling and attenuation of pro-survival and proliferative pathways [199]. Akt inhibition by capsaicin is frequently associated with modulation of upstream growth factor receptors, suppression of NF-κB and STAT3 signaling, induction of mitochondrial dysfunction, and activation of caspase-dependent apoptosis [200]. Capsaicin induces cell cycle arrest and apoptotic cell death and enhances sensitivity to chemotherapeutic agents and radiation [201]. Collectively, these findings position capsaicin as a plant-derived Akt pathway inhibitor with both chemopreventive and therapeutic relevance in Akt-dependent malignancies.

β-Elemene, a naturally occurring sesquiterpene extracted primarily from the rhizomes of *Curcuma wenyujin* and other *Curcuma* species [202], suppresses the PI3K/Akt pathway by downregulating Akt phosphorylation and kinase activity, leading to downstream inhibition of mTOR signaling and disruption of cancer cell survival, proliferation, and metabolic reprogramming [203]. Akt inhibition by β-elemene is closely associated with induction of mitochondrial-dependent apoptosis, modulation of Bcl-2 family proteins, and suppression of NF-κB-mediated transcription [204]. β-Elemene has shown to reverse chemoresistance and enhance sensitivity to chemotherapy and radiotherapy through attenuation of Akt signaling [205]. These properties highlight β-Elemene as a clinically relevant plant-derived Akt pathway inhibitor with both therapeutic and chemosensitizing potential.

### 5.2. Small Molecule Akt Inhibitors Derived from Animal Sources

Solenopsin is an alkaloid isolated from the venom secreted by *Solenopsis invicta*, commonly known as the fire ant. Solenopsin is a potent angiogenesis inhibitor. Its effect on angiogenesis is attributed to its ability to inhibit the PI3K signaling pathway upstream of PI3K. It also prevents phosphorylation of Akt and its physiologic substrate FOXO1α. It did not affect insulin-induced tyrosine phosphorylation of IRS1 but suppressed the PI3K activation and phosphorylation of its substrate including Akt at Thr-308 and Ser-473. It does not inhibit purified PI3K or PDK1. In vitro studies have demonstrated that it selectively inhibited Akt1 activity with an IC50 value of 5.0 to 10.0 μM in an ATP-competitive binding manner without affecting other protein kinases. Its attributes could be due to its structure, which has the long alkyl side chains resembling phospholipid ethers, and the positively charged amine group and alkyl chain that of Miltefosine and Perifosine [206] Table 2.

### 5.3. Small Molecule Akt Inhibitors Derived from Microbial Products:

#### 5.3.1. Bostrycin (Bos)

Bostrycin (Bos), an anthracenedione, was isolated from a broth culture of marine fungus *Bostrychonema alpestre.* Bos has been shown by many investigators to downregulate the PI3K/Akt pathway and thereby inhibit the proliferation of A549 cells. Mechanistically, Bos arrests cells in the G0/G1 phase of the cell cycle and upregulated microRNA-923 and microRNA-638 expression. Bos also downregulates PI3K-p110 and p-Akt, increased p27 activity, and inhibited cell proliferation. MicroRNA (miRNAs) activation and PI3K/Akt signaling inactivation induced cycle arrest and apoptosis [207]. Bos derivatives are more cytotoxic than the parent compound. Structural optimization of Bos has been shown to produce more effective derivatives with potent antitumor activity [208].

#### 5.3.2. Adriamycin Analog

Adriamycin Analog is an anthracenedione derived from marine endophytic fungus isolated from South China Sea. It induced apoptosis by blocking Akt activation in MCF-7 and MDA-MB-435. 1403P-3, an anthracenedione derivative, inhibited growth of MCF-7 and MDA-MB-435, with an IC50 of 9.5 and 7.6 µM, respectively. Apoptosis induced by 1403P-3 was confirmed by the presence of activated caspase-8 and -9 and cleaved PARP. It reduced phosphorylation of Akt in a dose- and time-dependent manner and induced apoptosis by blocking Akt activation in breast cancer cell [209].

#### 5.3.3. SZ-685C

SZ-685C is an anthraquinone derivative isolated from the mangrove endophytic fungus *Halorosellinia* sp. from South China Sea. It has anticancer activity and tumor suppressive effects. Studies have shown that it can inhibit Akt signaling in breast cancer cells and thus help in overcoming chemoresistance in the ADR-resistant cells MCF-7/ADR and MCF-7/Akt [210]. SZ-685C has also exhibited anti-proliferative activity in NFPA, RPCs and MMQ cell lines by inducing caspase-mediated apoptosis by decreasing the level of Akt [211].

#### 5.3.4. Wentilactone A

Wentilactone A, a compound isolated from marine endophytic fungi *Aspergillus wentii* EN-48 exhibited anti-cancer activity in small cell lung cancer (SCLC). It induced apoptosis by targeting AKR1C1 gene via the IGF-1R/IRS1/PI3K/AKT/Nrf2/FLIP/Caspase-3 signaling in SCLC. The compound is cytotoxic in certain tumor cell lines and exerts a significant activity in NCI-H446 and NCI-H460 [212]. It reduces phosphorylation of IGF-1R, IRS1, PI3K, Akt, Nrf2 and FLIP and increases cleaved caspase-3 expression [213].

#### 5.3.5. Thiocoraline

Thiocoraline, a depsipeptide bisintercalator antibiotic, from marine actinomycete *Micromonospora marina* L-13-ACM2-092, exhibited anticancer activity in MCF-7 at nanomolar concentrations via the PI3K/Akt/BCRP Signaling Pathway. It increases the Akt phosphorylation and hence its specific mechanism needs to be clearly elucidated [214].

#### 5.3.6. Lipopeptide Iturin A

Lipopeptide Iturin A is an ester peptide isolated from marine *Bacillus megaterium,* that inhibits Akt-mediated GSK3β and FoxO3α signaling and induces apoptosis in Breast Cancer Cells. Iturin A can sensitize drug-resistant breast tumor cells MDA-MB-468 and MDA-MB-231 against docetaxel by suppressing Akt activity [215].

#### 5.3.7. 1403P-3

1403P-3, a doxymycin analog isolated from the endophytic fungus No.1403, significantly inhibited MDA-MB-435 and MCF-7 cell lines with IC50 values of 7.6 and 9.5 μM, respectively. 1403P-3 increases activated caspase-8/-9 and PARP cleavage and significantly decreased phosphorylated Akt in a time- and dose-dependent manner [216]. Cytotoxic peptides B isebromoamide isolated from the marine cyanobacterium *Lyngbya* species inhibits ERK, Akt, mTOR and p70S6K phosphorylation in renal cell carcinoma and induces apoptosis. However, it has no effect on MEK, PDK1, PI3K, and EGF receptors [217].

#### 5.3.8. Xyloketal B

Xyloketal B, isolated from the fungus Xylaria inhibit TRPM7-regulated PI3K/Akt and MEK/ERK signaling pathways. It suppresses proliferation and migration of U251 cells. Five unique xyloketals A–E were isolated from the fungus *Xylaria* sp. (No. 2508). Xyloketals B–E are analogs of a ketal compound, xyloketal A. Xyloketal C rearranges into xyloketal B in DMSO at room temperature. Xyl B at 300 μM significantly decreases Akt and ERK1/2 phosphorylation and blocks TRPM7-mediated MEK/ERK and PI3K/Akt pathways in a reversible way and thus inhibits cell proliferation and migration of U251 glioblastoma [218].

#### 5.3.9. Diketopiperazine Demethoxyfumitremorgin C

It is isolated from marine *Aspergillus fumigatus* and inhibits PC3 cells by inducing apoptosis after exhaustion of MMP. This is due to inactivation of anti-apoptotic proteins including Ras, PI3K, Akt, Bcl-2 and Bcl-XL, and activation of pro-apoptotic Bax protein and caspase-3/-8/-9, causing PARP cleavage. Therefore, it suppresses PC3 cells through both intrinsic and extrinsic pathways [219,220].

#### 5.3.10. Lactoquinomycin

It is a pyrano-naphthoquinone antibiotic isolated from *Streptomyces* sp. LL-AF101 and selectively inhibits Akt [221]. It is a potent Akt inhibitor that has irreversible covalent interaction with two critical catalytic activation loops, Cys296 and Cys310, and is independent of PDK1-dependent Thr308 phosphorylation [221]. It involves S-alkylation with reduction in quinone ring to a hydroquinone and lactone ring opening. Lactoquinomycin inhibits phosphorylation of Akt and its downstream substrates GSK-3α/β, FKHRL1 and mTOR, as observed in various cancer cell lines. Similar Akt inhibitory activity has been observed with Frenolicin B (pyrano-naphthoquinone natural derivative), kalafungin and medermycin [222].

## 6. Conclusions

The study establishes the PI3K/Akt signaling cascade as a central oncogenic factor that integrates growth factor signaling, metabolic regulation, stress adaptation, and survival pathways to sustain malignant phenotypes across diverse cancer types. This review underscores that Akt-driven tumorigenesis cannot be explained by canonical phosphorylation events alone; rather, it is governed by a multilayered regulatory system having isoform-specific expression, dynamic subcellular localization, and an intricate network of post-translational modifications. Together, these mechanisms show signaling plasticity, enabling cancer cells to adapt to environmental stressors, evade therapeutic pressure, and acquire invasiveness, metabolic reprogramming, and stemness.

The Akt isoforms (AKT1, AKT2, and AKT3), despite their high sequence homology, show distinct biological functions depending on cellular context and tumor subtype. Isoform-specific differences in phosphorylation patterns, interacting partners, and intracellular trafficking critically influence downstream signaling outputs. Moreover, post-translational modifications, including phosphorylation, ubiquitination, acetylation, SUMOylation, O-GlcNAcylation, and redox-based modifications, act as molecular switches that fine-tune Akt activity independently of, or in coordination with, PI3K signaling. Such noncanonical regulatory routes explain how Akt signaling may persist in PTEN-deficient tumors or under PI3K inhibition, thereby contributing to acquired drug resistance.

The clinical translation of Akt inhibitors is limited by several factors. ATP-competitive inhibitors suffer from limited kinase selectivity and dose-limiting toxicities, while allosteric and covalent inhibitors, although more selective, have shown variable efficacy in advanced-stage clinical trials. These limitations highlight an indiscriminate inhibition of Akt signaling that disrupts essential physiological processes, particularly glucose homeostasis, underscoring the need for refined therapeutic strategies that achieve pathway suppression without unacceptable systemic toxicity. Consequently, future drug development efforts must prioritize isoform-selective inhibition, context-specific targeting, and modulation of Akt regulatory nodes beyond the conserved ATP-binding pocket.

Microbial-derived metabolites emerge as a good source of structurally diverse Akt modulators. The microbial compounds discussed in this review demonstrate that Akt activity can be attenuated through unconventional mechanisms, including interference with membrane recruitment, disruption of allosteric conformations, and modulation of downstream signaling complexes. Importantly, these agents often exhibit multitarget properties that may be advantageous in overcoming pathway redundancy and compensatory signaling. However, their translational potential remains limited by incomplete mechanistic characterization, limited data on Akt isoform specificity, and insufficient evaluation in in vivo models.

Effective targeting of Akt-driven cancers will require a precision oncology approach that accounts for the biological complexity of Akt signaling. This includes classification of tumors based on their dependence on specific Akt isoforms, patterns of post-translational modifications, and subcellular localization of Akt. Such molecular stratification should be integrated with rational drug design and well-planned combination therapies to improve efficacy and minimize toxicity. The use of advanced preclinical models, including patient-derived xenografts and organoids, will be essential for accurately assessing therapeutic responses and identifying reliable biomarkers that predict treatment outcome.

## Figures and Tables

**Table 2:** List of Akt inhibitors with their structure and mechanism of action.

Name of the Akt inhibitor	Structure	Formula and Molecular Weight	Solubility	Targeted Domain	References
Borussertib	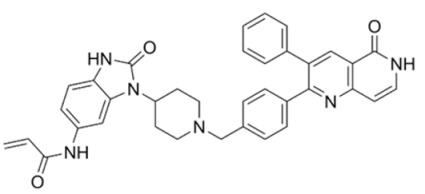	C_36_H_32_N_6_O_3_ 596.68	DMSO (25 mg/mL, i.e., 41.9 mM)	Allosteric inhibitor, which forms a covalent bond with Cys296. Binds to the interdomain pocket located between PH and Catalytic domains	[93]
NL-71-101	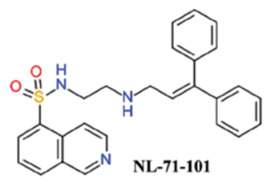	C_26_H_24_N_4_O_2_S 456.56	DMSO (20 mg/mL, i.e., 43.8 mM)	PH domain and ATP binding site	[95]
Aminofurazan (GSK690693)	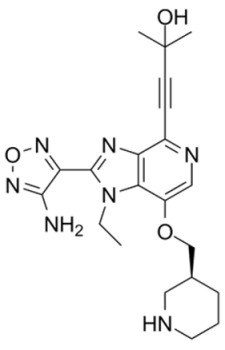	C_21_H_27_N_7_O_3_ 425.5	DMSO and DMF (25 mg/mL, i.e., 58.75 mM)	ATP binding site of Akt	[112,113,114]
CCT128930	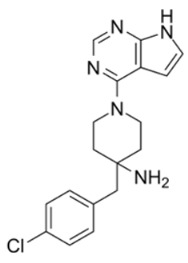	C_18_H_20_C_l_N_5_ 341.84	DMSO (33.33 mg/mL., i.e., 97.5 mM)	ATP binding site of Akt	[104]
Ipatasertib (GDC0068)	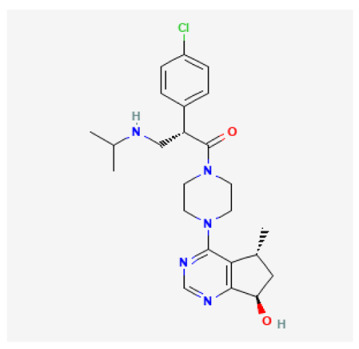	C_24_H_32_C_l_N_5_O_2_ 458.0	DMSO (50 mg/mL, i.e., 109.1 mM) and DMF (25 mg/mL, i.e., 54.58 mM)	ATP binding site of Akt	[109]
Afuresertib (GSK2110183)	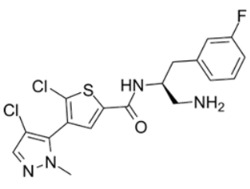	C_18_H_17_C_l2_FN_4_OS 427.32	DMSO (90 mg/mL, i.e., 210.6 mM) DMF (50 mg/mL, i.e., 117.0 mM) Ethanol (50 mg/mL, i.e., 117.0 mM)	ATP binding site of Akt	[119]
Uprosertib (GSK2141795)	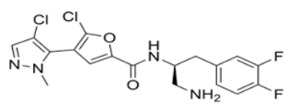	C_18_H_16_C_l2_F_2_N_4_O_2_ 429.25	DMSO (50 mg/mL, i.e., 116.48 mM)	ATP binding site of Akt	[119]
MK-2206 dihydrochloride	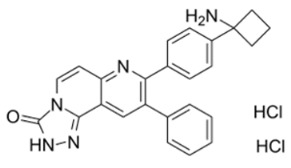	C_25_H_23_C_l2_N_5_O 480.39	DMSO 12.5 mg/mL, i.e., 26.02 mM	Non-competitive inhibitor of Akt. Binds at the interface of the PH and Catalytic domains	[134]
Edelfosine (Et-18-OCH3)	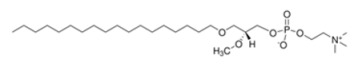	C_27_H_58_NO_6_P 523.73	DMSO 25 mg/mL, i.e., 47.73 mM	Selectively aggregates cell death receptor Fas in membrane rafts and interfere with phosphatidyl choline synthesis	[140]
Ilmofosine	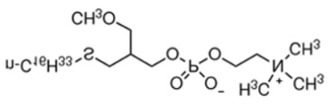	C_26_H_56_NO_5_PS 525.77	Water 10 mg/mL, i.e., 19.02 mM	Inhibits the Akt activation by interacting with PH domain	[141]
Miltefosine	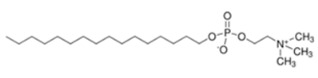	C_21_H_46_NO_4_P 407.57	Water 10 mg/mL, i.e., 24.53 mM	Inhibits the Akt activation by interacting with PH domain	[145]
Perifosine	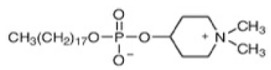	C_25_H_52_NO_4_P 461.66	Water 10 mg/mL, i.e., 21.66 mM	Inhibits the Akt activation by interacting with PH domain	[145]
Erufosine	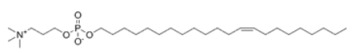	C_28_H_58_NO_4_P 503.7	Water 10 mg/mL i.e., 19.85 mM	Inhibits the Akt activation by interacting with PH domain	[146,147]
API-2 (Triciribine)	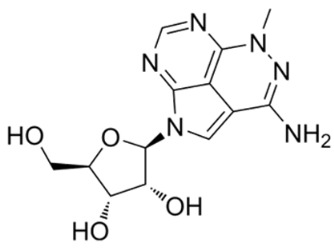	C_13_H_16_N_6_O_4_ 320.3	DMSO 100 mg/mL 312.21 mM	Binds to PH domain and prevents the translocation of Akt to cell membrane	[159,160]
Apigenin	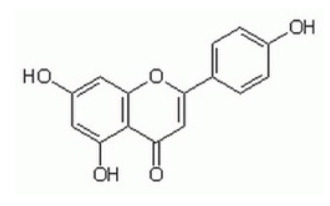	C_15_H_10_O_5_ 270.24	DMSO 10 mg/mL 37.0 mM	Inhibits phosphorylation of Akt by targeting upstream kinases	[172]
Curcumin	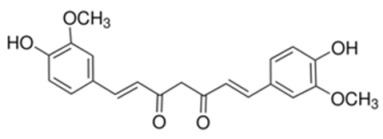	C_21_H_20_O_6_ 368.38	DMSO 11 mg/mL 29.86 mM	Inhibits phosphorylation of Akt by targeting upstream kinases	[173]
Fisetin	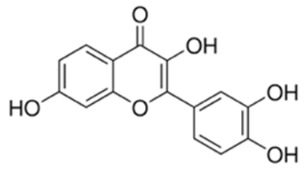	C_15_H_10_O_6_ 286.24	DMSO 2.0 mg/mL 6.98 mM	Inhibits phosphorylation of Akt by targeting upstream kinases	[174,175,176]
Indole-3-carbinol	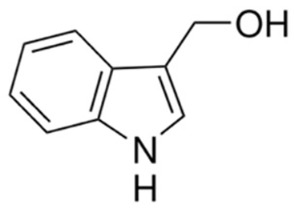	C_9_H_9_NO 147.17	DMSO 3.0 mg/mL 20.38 mM Ethanol 10 mg/mL 67.94 mM	Inhibits phosphorylation of Akt by targeting upstream kinases	[177,178,179,180]
Resveratrol	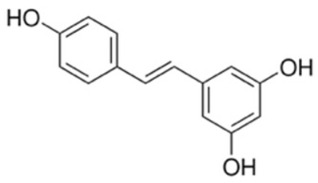	C_14_H_12_O_3_ 228.24	Acetone 50 mg/mL 219.06 mM	Elevates PTEN expression and decreases phosphorylation of Akt	[181,182,184]
Caffeine	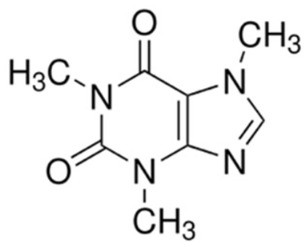	C_8_H_10_N_4_O_2_ 194.19	Water 20 mg/mL at 25 °C 102.99 mM	Inhibits phosphorylation of Akt by targeting upstream kinases	[185,186,187]
Epigallocatechin gallate	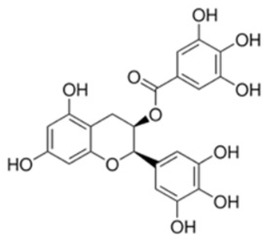	C_22_H_18_O_11_ 458.37	Water 25 mg/mL 54.54 mM	ATP competitive inhibitor of upstream PI3K. It decreases phosphorylation of Akt	[188,189,190,191]
Celastrol	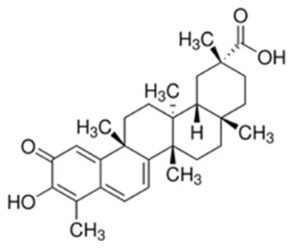	C_29_H_38_O_4_ 450.61	DMSO 10 mg/mL 22.19 mM	Elevates PTEN expression and decreases phosphorylation of Akt	[192,193]
Butein	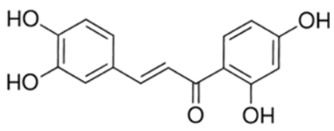	C_15_H_12_O_5_ 272.25	DMSO 20 mg/mL 73.46 mM	Decreases phosphorylation of Akt	[194,195,196,197]
Capsaicin	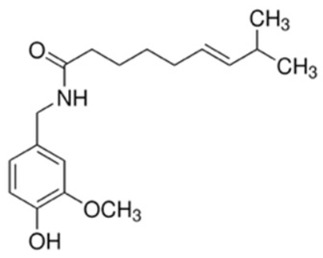	C_18_H_27_NO_3_ 305.41	DMSO 30 mg/mL 98.22 mM	Decreases phosphorylation of Akt	[198,199,200,201]
Beta-Elemene	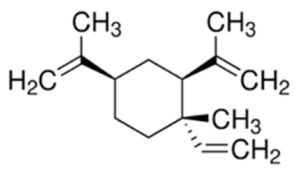	C_15_H_24_ 204.35	DMSO 30 mg/mL 146.80	Decreases phosphorylation of Akt	[202,203,204,205]

## Data Availability

No new data were created or analyzed in this study.

## Abbreviations

The following abbreviations are used in this manuscript:

4E-BP1	Eukaryotic initiation factor 4E (eIF4E)-binding protein 1
5-FU	5-Fluorouracil
Akt	AKR Mouse Strain Transforming or Thymoma
ALPs	Alkylphospholipids
AML	Acute Myeloid Leukemia
AMPK	AMP-activated protein kinase
ARNO	Arf Nucleotide-binding site Opener
ATM	Ataxia-Telangiectasia Mutated
ATP	Adenosine triphosphate
ATR	ATM and Rad3-related
BAD	Bcl-2 Associated Agonist of Cell Death
BB Barrier	Blood–Brain Barrier
BCL2	B-cell lymphoma 2
BCRP	Breast Cancer Resistance Protein
BL2	Basal-like 2
CBP	CREB-binding protein
CDK	Cyclin-Dependent Kinase
CIP1	CDK-Interacting Protein 1
CNE2	Nasopharyngeal Carcinoma Epithelial cell line
CRCs	Colorectal Cancers
CYP3A4	Cytochrome P450 3A4
DMSO	Dimethyl Sulfoxide
DNA	Deoxyribonucleic acid
DNA-PK	DNA-dependent protein kinase
EGCG	Epigallocatechin gallate
EGF	Epidermal Growth Factor
EGFR	Epidermal Growth Factor Receptor signaling
EMT	Epithelial–Mesenchymal Transition
ERK	Extracellular signal-Regulated Kinase
ErPC	Erucylphosphocholine
FKHR	Forkhead in Rhabdomyosarcoma
FLIP	FLICE-like-inhibitory-protein
FOXO	Forkhead box O
GlcNAc	N-Acetylglucosamine
GRP1	General receptor for 3-phosphoinositides 1
GSK3	Glycogen Synthase Kinase 3
HATs	Histone Acetyltransferases
HBP	Hexosamine Biosynthetic Pathway
HDACs	Histone Deacetylase
HIF	Hypoxia-Inducible Factor
HM	Hydrophobic Motif
HR+	Hormone Receptor Positive
HSP90	Heat Shock Protein 90
IGF-1R	Insulin-like Growth Factor-1 Receptor
IKB Kinase-E	I kappa B kinase epsilon
IKK	IκB kinase
IL-6	Interleukin 6
IL-8	Interleukin 8
ILK	Integrin-Linked Kinase
INPP4B	Inositol polyphosphate 4-phosphatase type II
IRS-1	Insulin Receptor Substrate 1
MAPK	Mitogen-Activated Protein Kinase
MDA-MB-231	MD Anderson-Metastatic Breast-231
MDM2	Murine Double Minute 2
MMPs	Matrix Metalloproteinases
mTOR	Mechanistic Target of Rapamycin
NCI-H460	National Cancer Institute-Human-460
NEDD4-1	Neural precursor cell expressed developmentally down-regulated 4-1
NF-κB	Nuclear Factor kappa-light-chain-enhancer of activated B cells
NLS	Nuclear Localization Signal
NRF2	Nuclear factor erythroid 2-related factor 2
OGA	O-GlcNAcase
OGT	O-GlcNAc Transferase
PARP	Poly(ADP-ribose) polymerase
PDK-1	Pyruvate Dehydrogenase Kinase-1
PDX	Patient Derived Xenograft
PH Domain	Pleckstrin Homology Domain
PHLPP	PH domain Leucine-rich repeat Protein Phosphatase
PI3K	Phosphatidylinositol 3-kinase
PIP3	Phosphatidylinositol (3,4,5)-trisphosphate
PIT1	Pituitary-specific transcription factor 1
PKB	Protein Kinase B
PKCβII	Protein Kinase C beta II
PRAS40	Proline-rich Akt Substrate of 40 kDa
PTEN	Phosphatase and Tensin Homolog deleted on Chromosome 10
PTK	Protein Tyrosine Kinase
PTMs	Post-translational modifications
RNA	Ribonucleic acid
ROS	Reactive Oxygen Species
RTK	Receptor Tyrosine Kinase
S6K1	S6 Kinase 1
SCLC	Small Cell Lung Cancer
Skp2	S-phase kinase-associated protein 2
STAT1	Signal Transducer and Activator of Transcription 1
SUMO	Small Ubiquitin-like Modifier
SW	Swertiamarin
TBK1	TANK-binding kinase 1
TCN-P	Triciribine Phosphate
TM	Turn Motif
TNBC	Triple-Negative Breast Cancer
TNF	Tumor Necrosis Factor
TRAF6	Tumor Necrosis Factor Receptor-Associated Factor 6
TRPM7	Transient Receptor Potential Cation Channel, Subfamily M, Member 7
TSC	Tuberous sclerosis complex
VEGF	Vascular Endothelial Growth Factor
WAF1	Wildetype p53-Activated Fragment 1
